# The Spatiotemporal Control of Zygotic Genome Activation

**DOI:** 10.1016/j.isci.2019.06.013

**Published:** 2019-06-11

**Authors:** George E. Gentsch, Nick D.L. Owens, James C. Smith

**Affiliations:** 1 Developmental Biology Laboratory, Francis Crick Institute, London NW1 1AT, UK; 2 Department of Stem Cell and Developmental Biology, Pasteur Institute, Paris 75015, France

**Keywords:** Developmental Genetics, Developmental Biology, Embryology, Omics

## Abstract

One of the earliest and most significant events in embryonic development is zygotic genome activation (ZGA). In several species, bulk transcription begins at the midblastula transition (MBT) when, after a certain number of cleavages, the embryo attains a particular nuclear-to-cytoplasmic (N/C) ratio, maternal repressors become sufficiently diluted, and the cell cycle slows down. Here we resolve the frog ZGA in time and space by profiling RNA polymerase II (RNAPII) engagement and its transcriptional readout. We detect a gradual increase in both the quantity and the length of RNAPII elongation before the MBT, revealing that >1,000 zygotic genes disregard the N/C timer for their activation and that the sizes of newly transcribed genes are not necessarily constrained by cell cycle duration. We also find that Wnt, Nodal, and BMP signaling together generate most of the spatiotemporal dynamics of regional ZGA, directing the formation of orthogonal body axes and proportionate germ layers.

## Highlights

• Temporal RNAPII profiling shows gradual activation of zygotic genome in frog embryo
• Quantity and size of RNAPII-transcribed genes increase before cell cycle slows down
• More than 1,000 genes are activated before the midblastula transition
• Most regional activation of zygotic genome depends on Wnt, Nodal, and BMP signaling

## Graphical Abstract

**Figure:**
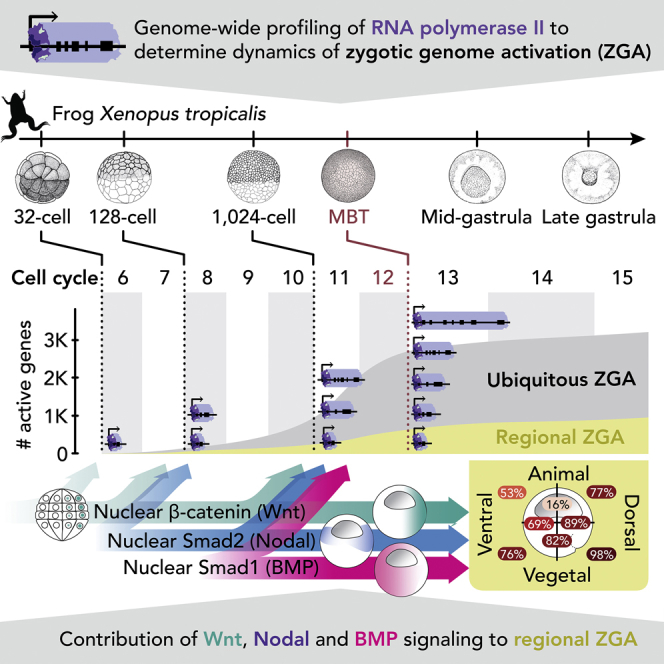

## Introduction

The genomes of multicellular organisms are transcriptionally silent at the time of fertilization, and the events of early development, including zygotic (also known as embryonic) genome activation (ZGA), are directed by maternal gene products (De Iaco et al., 2019, Eckersley-Maslin et al., 2019, Gentsch et al., 2018b, Lee et al., 2013, Leichsenring et al., 2013, Liang et al., 2008). The number of cell cycles after which ZGA becomes essential for development (at which embryos arrest if transcription is inhibited) is highly reproducible within each species. In the zebrafish, the frog *Xenopus*, and the fruit fly *Drosophila*, this occurs after 10, 12, and 13 cell cycles, respectively, at the so-called midblastula transition (MBT) (Blythe and Wieschaus, 2015, Gentsch et al., 2018b, Kane and Kimmel, 1993, Newport and Kirschner, 1982a). Early development in these species occurs with no gain in cytoplasmic volume, and studies in *Xenopus* suggest that ZGA is triggered at a particular nuclear-to-cytoplasmic (N/C) ratio, when the increasing amount of nuclear DNA titrates out maternally deposited repressors (Newport and Kirschner, 1982b). Slower-developing mammalian embryos show major waves of RNA polymerase II (RNAPII)-mediated transcription as early as the two-cell stage in mice (Bolton et al., 1984, Hamatani et al., 2004) and four- to eight-cell stage in humans (Braude et al., 1988, Vassena et al., 2011). This occurs days before the formation of the blastocyst, which, like the blastula, contains the pluripotent cells that form the embryo proper.

In *Xenopus*, ZGA is associated with changes in cell behavior after the MBT. First, rapid and nearly synchronous cell cleavages give way to longer and asynchronous cell divisions (Anderson et al., 2017, Newport and Kirschner, 1982a). Second, embryonic cells acquire the ability to respond to inductive signaling (Gentsch et al., 2018b), causing them to become motile, to establish dorsoventral patterning, and to contribute to one or two of the three germ layers (endoderm, mesoderm, and ectoderm). These germ layers emerge first during gastrulation and are the primordia of all organs. Third, embryos show accelerated degradation of maternal RNA, and fourth, cells gain apoptotic (Stack and Newport, 1997) and immunogenic (Gentsch et al., 2018a) capabilities.

Although large-scale ZGA occurs at the MBT, some genes escape the repressive environment of the early embryo and nascent transcripts can be detected in *Xenopus* during rapid cleavage stages. For example, primary microRNA transcripts of the polycistronic MIR-427 gene (Lund et al., 2009) are detectable in *Xenopus tropicalis* after just three cell divisions (Owens et al., 2016). MIR-427, like its zebrafish equivalent MIR-430, is activated at early stages by the synergistic and pioneering activities of maternal members of the SoxB1 and Pou5F (Oct4) transcription factor (TF) families (Gentsch et al., 2018b, Heyn et al., 2014, Lee et al., 2013). These core pluripotency TFs, represented by Sox3 and Pou5f3 in *Xenopus*, are characterized by ubiquitous and high translation frequencies in pre-MBT embryos (Gentsch et al., 2018b, Lee et al., 2013). Zygotic transcription of the Nodal-encoding genes *nodal3/5/6*, and of the homeobox genes *siamois1/2*, is initiated by nuclear β-catenin as early as the 32-cell stage (Owens et al., 2016, Skirkanich et al., 2011, Yang et al., 2002).

Although miR-427 (and miR-430 in zebrafish) contributes to the clearance of maternal RNA (Giraldez et al., 2006, Lund et al., 2009), *nodal* and *siamois* genes initiate the formation of the germ layers and body axes (Agius et al., 2000, Lemaire et al., 1995). All these genes, and other early-activated genes in *Drosophila*, *Xenopus*, and zebrafish, have coding sequences of <1 kb and either no introns or just a few (Heyn et al., 2014). It has been suggested that the early rapid cell cycles cause the DNA replication machinery to interfere with the transcription of larger genes (Shermoen and O'Farrell, 1991), a suggestion supported, to date, by the profiling of nascent transcripts (Heyn et al., 2014). We note, however, that the detection and temporal resolution of de novo transcription can be particularly challenging for genes that have both maternal and zygotic transcripts.

Here we use the continuous occupancy of RNAPII along gene bodies as a method to record ZGA. In contrast to transcript profiling techniques, this method (1) directly determines the activity of every gene; (2) is independent of metabolic labeling (Heyn et al., 2014) and of any gene feature such as introns (Lee et al., 2013), single nucleotide polymorphisms (Harvey et al., 2013, Lott et al., 2011), or transcript half-lives; and (3) circumvents difficulties in detecting nascent transcripts in a large pool of maternal transcripts. By combining these data with the profiling of the transcriptome along the primary body axes (Blitz et al., 2017), we resolve ZGA in time and space for wild-type and various loss-of-function embryos. We provide evidence that runs counter to our original understanding of the cell cycle or of the N/C ratio in constraining gene expression before MBT. And finally, we show how signaling initiates and coordinates spatiotemporal ZGA in the *Xenopus* embryo.

## Results

### RNAPII Profiling Reveals Exponential ZGA before MBT

**Figure 1:**
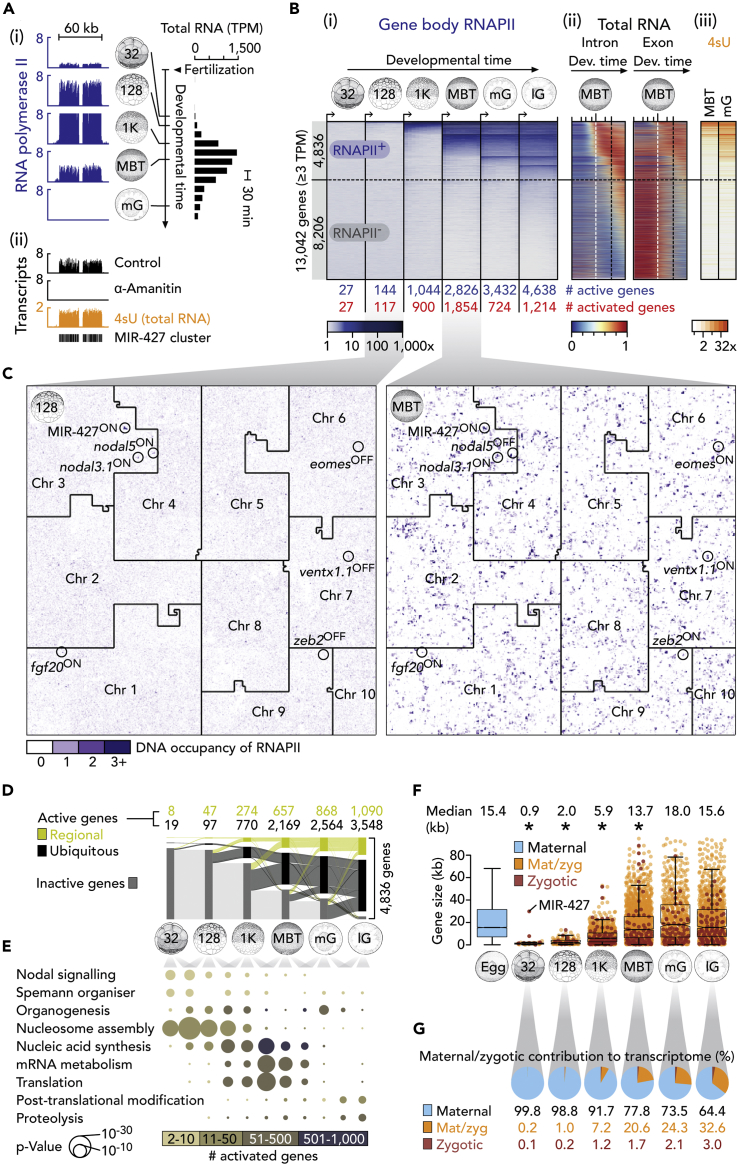
Dynamics and Architecture of ZGA in *X. tropicalis* (A) (i) Genome-wide profiling of RNAPII and total RNA (Owens et al., 2016) to determine temporal ZGA dynamics. (ii) Complementary approach to detect transcriptionally active genes by α-amanitin-induced loss and 4sU enrichment of nascent (zygotic) transcripts. (B) Progression of ZGA from the 32-cell to the late gastrula stage based on (i) whole gene body (full-length) occupancy of RNAPII (i.e., RNAPII was enriched across entire gene bodies; see Transparent Methods). Co-aligned: (ii) High time-resolution of total RNA, separated by intron- and exon-derived signals, from fertilization to the late gastrula stage, and (iii) enrichments of 4sU-tagged RNA at the MBT and the mid-gastrula stage. Numbers below RNAPII heatmap represent counts of active (blue) and activated (red) genes at the indicated developmental stages. The horizontal dotted line separates RNAPII-engaged (RNAPII^+^) from non-engaged (RNAPII^−^) genes as detected until the late gastrula stage. The vertical dotted lines in the total RNA plots indicate the developmental time points of the MBT (white) and the late gastrula stage (black), respectively. (C) 2D space-filling (Hilbert) curves showing RNAPII recruitment to chromosomes (Chr) at the 128-cell stage and the MBT. A few zygotic genes are highlighted as being active (ON) or not (OFF) based on their engagement with RNAPII. (D) Alluvial diagram of spatiotemporal ZGA. Tissue specificity inferred from regional transcript enrichments along the animal-vegetal or the dorsoventral axes or both (Blitz et al., 2017). (E) ZGA-associated enrichment of biological processes. The statistical significance of enrichment (hypergeometric p-value) an the number of activated genes associated with each biological process are visualized by bubble size and color, respectively. (F) Beeswarm boxplots of maternal and/or zygotic gene sizes. *, p < 1.9 × 10^−7^ (Wilcoxon rank-sum test against maternal and post-MBT activated genes); r_effect_ 0.06 (“MBT” vs “Egg”) - 0.48 (“128” vs “mG”). (G) Maternal/zygotic contribution to the transcriptome deduced from full-length RNAPII occupancy and maternally inherited RNA. Abbreviations: 32, 32-cell; 128, 128-cell; 1K, 1,024-cell; MBT, midblastula transition; mG, mid-gastrula; lG, late gastrula; 4sU, 4-thiouridine; Mdn, median; TPM, transcripts per million. See also Figure S1 and Tables S1 and S2.

In an effort to resolve the progression of ZGA, we profiled chromatin for RNAPII engagement on hand-sorted *X. tropicalis* embryos over six developmental stages from the 32-cell to the late gastrula stage (Figures 1A and 1B). RNAPII was localized on the genome by chromatin immunoprecipitation followed by deep sequencing (ChIP-seq). We complemented RNAPII profiling with high time-resolution transcriptomics (Owens et al., 2016) counting both exonic and intronic RNA at 30-min intervals from fertilization to the late gastrula stage (Figures 1A, 1B, S1A, and S1B). For both maternal and zygotic genes, the detection threshold was set to ≥3 transcripts per million (TPM) averaged over any 1-h window during this developmental period to avoid genes with general low-level expression. This restricted the analysis to 13,042 genes (Figure 1B). These genes were considered active when we detected simultaneously RNAPII enrichment along their full length (see Transparent Methods) as well as the presence of the corresponding transcripts. In doing so, we used a low threshold of ≥0.1 TPM so as not to miss the onset of gene transcription. RNAPII-guided ZGA profiling was verified in part by active post-translational histone marks (Hontelez et al., 2015) and by differential expression methods aiming at detecting nascent transcripts. Thus, zygotic transcript depletion (by blocking RNAPII elongation with α-amanitin) (Gentsch et al., 2018b) or enrichment (by selecting 4-thiouridine [4sU]-tagged transcripts at the MBT and the mid-gastrula stage) showed substantial overlaps and positive correlations with RNAPII-covered genes (Figures 1A, 1B, S1C, and S1D and Tables S1 and S2).

This analysis revealed an exponential ZGA before the MBT with 27, 144, and 1,044 active genes after 5 (32-cell, ∼2.5 hpf), 7 (128-cell, ∼3 hpf) and 10 (1,024-cell, ∼4 hpf) cell cycles, respectively. Gene activation reached its peak at the MBT (∼4.5 hpf), with 1,854 newly activated genes, before dropping to 724 genes at the early to mid-gastrula stage (∼7.5 hpf) and increasing again to 1,214 genes toward the end of gastrulation (∼10 hpf) (Figures 1B, 1C, and S1E and Table S2). The dramatic increase in transcriptional activity that occurs in the 1.5 h between the 128-cell stage and the MBT can be illustrated by Hilbert curves (Figure 1C), which provide a genome-wide overview of RNAPII enrichment by folding chromosomes into two-dimensional space-filling and position-preserving plots (Gu et al., 2016). Although most zygotic genes remain active beyond the mid-gastrula stage, 197 (including *siamois2* [*sia2*], *nodal5* and *znf470*) of the 4,836 zygotic genes (∼4%) are deactivated within ∼6 h of development (Figures 1C, S1F, and S1G). Slightly less than one-third of the activated genes were differentially expressed along either or both of the animal-vegetal and dorsoventral axes (Figures 1D and S1F).

The temporal order of enriched biological processes supported by ZGA matched the regulatory flow of gene expression, starting with nucleosome assembly, nucleic acid synthesis, mRNA metabolism and production, post-translational modification, and degradation of proteins (Figure 1E). The earliest transcriptional engagement, beginning at the 32- to 128-cell stages, was detected in gene clusters of tens to hundreds of kilobases (Figures 1A, 1C, and S1H–S1J). These clusters featured close relatives of the same genes, some of which are critical to Nodal signaling (Nodal ligands), the formation of the Spemann organizer (Siamois homeobox transcription factors), nucleosome assembly (histones), mRNA decay (MIR-427), and ongoing gene regulation (zinc finger transcription factors with on average 10 Cys2-His2 [C2H2] domains; Figure S1J). These earliest activated genes were shorter and encoded smaller proteins than those within the maternal pool or those that are activated post-MBT (Figures 1F and S1K). The non-coding features that contributed most to the differences in length were the 3′ UTRs and introns (Figure S1K).

We noted that the shorter zygotic genes observed before the MBT did not correlate strictly with the time constraints imposed by short cell cycles. We detected increasing and wider spread of de novo recruitment of RNAPII before the MBT, when cleavages continue to occur at rapid and near-constant pace (Figures 1F and S1K). During this period, the median length of activated genes (and their coding sequences) increases from ∼0.9 kb (∼0.4 kb) to ∼5.9 kb (∼0.9 kb). However, it was not until after the MBT that the overall architecture of zygotic and maternal genes became indistinguishable (Figures 1F and S1K). Temporal comparison of RNAPII engagement and total RNA profiling suggested that the zygotic contribution to the transcriptome (as calculated by the number of zygotic genes divided by the number of genes with ≥0.1 TPM maternal transcripts averaged over the first hour post-fertilization when the entire zygotic genome is still transcriptionally inactive) rose within seven cell cycles from ∼0.2% at the 32-cell stage to ∼22% at the MBT (Figure 1G). Further maternal degradation and more moderate transcriptional engagement extended the zygotic contribution to about one-third of the transcriptome by the late gastrula stage. Maternal transcripts (≥0.1 TPM, see earlier text) were detected for ∼67% of newly activated genes (18 of 27 genes) at the 32-cell stage, ∼85% (99/117) at the 128-cell stage, ∼87% (780/900) at the 1,024-cell stage, ∼95% (1,754/1,854) at the MBT, ∼89% (644/724) at the mid-gastrula stage, and ∼90% (1,094/1,214) at the late gastrula stage (Table S2). Altogether ∼91% (4,389/4,836) of newly activated genes have ≥0.1 TPM maternal contribution.

### Wnt, Nodal, and BMP Signals Are Key Drivers of Regional ZGA

**Figure 2:**
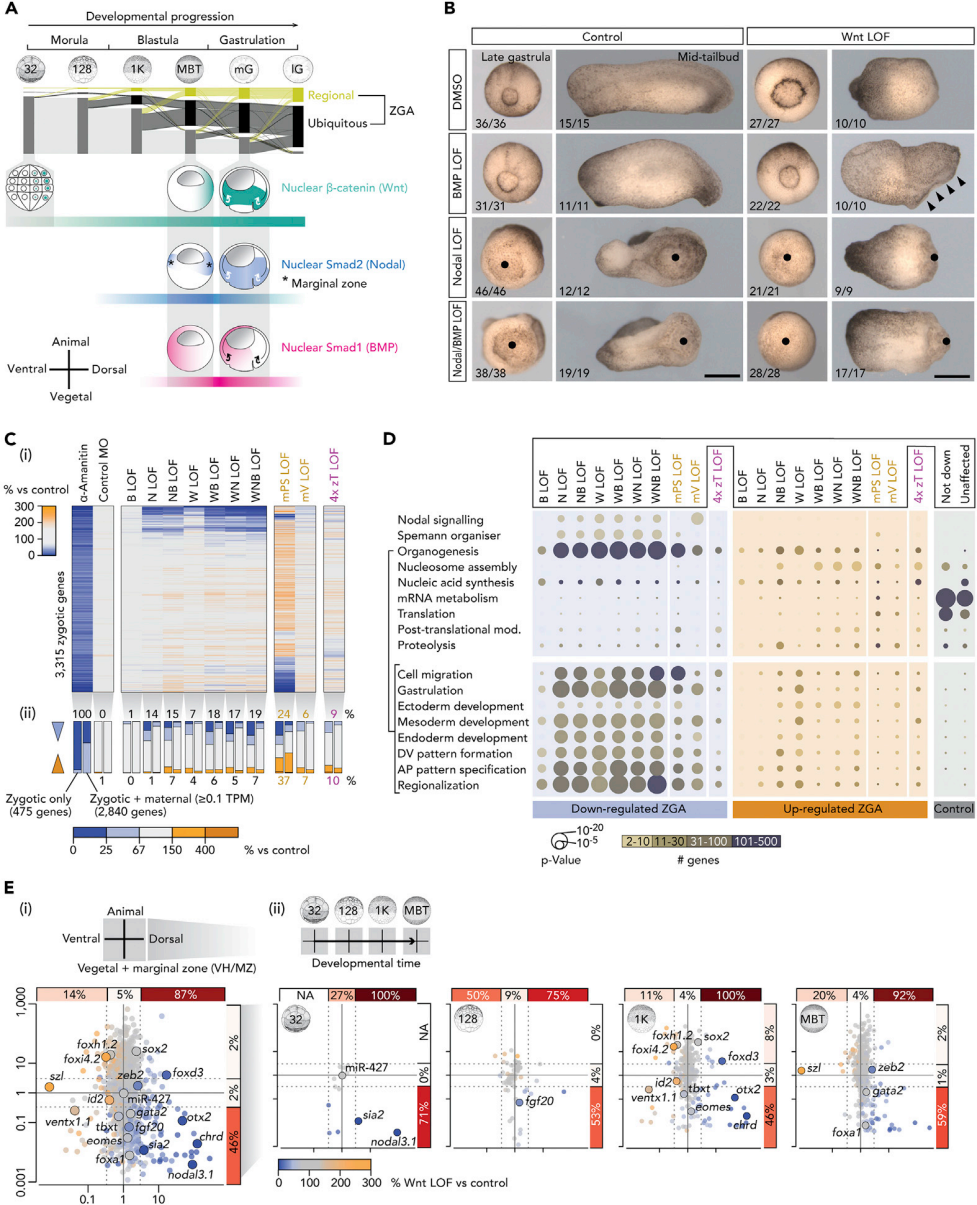
Spatiotemporal ZGA Regulated by Canonical Wnt, Nodal, and BMP Signals (A) Spatiotemporal ZGA and nuclear localization of signal mediators β-catenin (canonical Wnt), Smad2 (Nodal), and Smad1 (BMP) (Faure et al., 2000, Larabell et al., 1997, Schohl and Fagotto, 2002) from the 32-cell to the late gastrula stage. (B) Morphological phenotypes of single and combined signal LOFs at the late gastrula and the mid-tail bud stage. Left (“control”) pictures are taken from Gentsch et al. (2018b). Bullet points, failed blastopore formation. Arrowheads, excessive neural fold formation. Scale bar, 0.5 mm. (C) Heatmap (i) and bar graph summary (ii) of ZGA mis-regulated in various LOF embryos: α-amanitin, positive control; control MO, negative control. Abbreviations: B, BMP; N, Nodal; W, canonical Wnt; mPS, maternal Pou5f3/Sox3; mV, maternal VegT; 4x zT, four zygotic T-box TFs (zygotic VegT, Eomes, Tbxt and Tbxt.2). (D) Biological processes enriched with mis-regulated and control (not down-regulated or unaffected by the loss of maternal TFs or signaling) sets of zygotic genes under indicated LOFs. The statistical significance of enrichment (hypergeometric p-value) and the number of zygotic genes are visualized by bubble size and color, respectively. (E) Summary (i) and temporal resolution (ii) of Wnt LOF effects on regional ZGA. Percentages only refer to the down-regulated genes among all zygotic genes with similar expression ratios along the animal-vegetal or the dorsoventral axes. See also Figure S2, Tables S1 and S3, and Video S1.

We next sought to investigate the single and combined effects of different inductive signals on the spatiotemporal dynamics of ZGA. The early vertebrate embryo employs canonical Wnt, Nodal, and BMP signals and their key transcriptional effectors β-catenin, Smad2, and Smad1, respectively, to establish the primary body axes and the three germ layers (reviewed by Arnold and Robertson, 2009 and Kimelman, 2006). In *Xenopus*, β-catenin first translocates to the nuclei of dorsal blastomeres at the 32-cell stage (Larabell et al., 1997, Schneider et al., 1996) (Figure 2A). After the MBT, zygotic Wnt8a causes more nuclear β-catenin to accumulate around the forming blastopore lip (Christian and Moon, 1993, Schohl and Fagotto, 2002). The nuclear translocation of Smad1 and Smad2 is triggered around the MBT by various BMP and Nodal ligands. Nuclear Smad1 is primarily detected on the ventral side and the blastocoel roof of the embryo, whereas nuclear Smad2 is detected within the vegetal hemisphere (VH) and the marginal zone (MZ) (Faure et al., 2000, Schohl and Fagotto, 2002) (Figure 2A).

In an effort to inhibit canonical Wnt signaling, we injected into the *X. tropicalis* zygote a previously validated antisense morpholino oligonucleotide (MO), which interferes with β-catenin protein synthesis by annealing to the translation start codon (Heasman et al., 2000). Nodal and BMP signals were selectively blocked by incubating dejellied embryos from the eight-cell stage in the cell-permeable inhibitors SB431542 (Ho et al., 2006, Inman et al., 2002) and LDN193189 (Cuny et al., 2008, Young et al., 2017), respectively. The morphological phenotypes of these single loss-of-function (LOF) treatments were consistent with previous observations and ranged from impaired axial elongation causing the loss of tail structures (BMP, Reversade et al., 2005) to severe gastrulation defects (Wnt, Heasman et al., 2000, and Nodal, Ho et al., 2006) as shown in Figure 2B. Briefly, Nodal LOF impaired blastopore lip formation and bulk tissue movements of gastrulation (bullet points in Figure 2B). However, it did not preclude subsequent elongation of the antero-posterior axis. By contrast, Wnt LOF embryos underwent gastrulation (albeit delayed and more circumferentially rather than in a dorsal to ventral wave) but failed to form an antero-posterior axis, with both head and tail being absent. With respect to the joint effects of Wnt, Nodal, and BMP signaling, most dual and triple LOFs combined their individual morphological defects such that, for example, Wnt/Nodal LOF resulted in the complete loss of gastrulation and axial elongation. By contrast, Wnt/BMP LOF produced defects such as non-fusing neural folds (arrowheads in Figure 2B), structures that were either absent in Wnt LOF embryos or normal in BMP LOF embryos.

Changes to ZGA caused by the single or combined LOF of Wnt, Nodal, and/or BMP were then determined at the late blastula stage on a transcriptome-wide scale using deep RNA sequencing (RNA-seq). Analysis was limited to the 3,315 zygotic genes for which spatiotemporal expression data are available (Blitz et al., 2017, Owens et al., 2016) and where reduced expression (≥50% loss of exonic and/or intronic transcript counts, false discovery rate [FDR] ≤10%) could be detected in α-amanitin-injected embryos (Figure 2C and Table S3) (Gentsch et al., 2018b). α-Amanitin-mediated inhibition of RNAPII elongation impedes the morphogenetic tissue movements of gastrulation and ultimately leads to early embryonic death (Gentsch et al., 2018b). Spatial gene expression patterns were inferred from experiments comparing the transcriptomes of embryos dissected along their animal-vegetal and dorsoventral axes (Blitz et al., 2017); we did not include the left-right axis because there were no significant differences in gene expression across this axis at the gastrula stage (Blitz et al., 2017). The signal-mediated transcriptional effects (1.5-fold change from control RNA level) on zygotic genes, 86% (2,840/3,315) of which have ≥0.1 TPM maternal contribution, ranged from ∼1.5% (∼1.3% down and ∼0.2% up) to ∼26% (∼19% down and 7% up) for single BMP LOF and triple Wnt/Nodal/BMP LOFs, respectively (Figure 2C). As expected, the transcript levels of genes whose expression is solely zygotic were more strongly affected than those of zygotic genes with maternally contributed transcripts (Figures 2C and 4A). The extent of ZGA mis-regulation largely reflected the severity of the resulting morphological phenotypes at the late gastrula and the mid-tail bud stage (Figures 2B and 2C).

In comparison, the LOFs of critical maternal TFs like Pou5f3/Sox3 or VegT (Gentsch et al., 2018b) caused the mis-regulation of 61% (∼24% down and ∼37% up) and 13% (∼6% down and ∼7% up) of zygotic genes, respectively. The LOFs of four zygotic T-box TFs (zVegT, Eomes, Tbxt, and Tbxt.2), all of which require Nodal signaling for their expression, caused slight mis-regulation in 19% (∼9% down and ∼10% up) of the zygotic genes as detected over three consecutive developmental time points during gastrulation (Table S3). Among the ZGA-enriched biological functions (Figure 1D), Wnt, Nodal, and BMP signals, like maternal Pou5f3/Sox3 and VegT, strongly affected zygotic genes associated with cell migration, gastrulation, dorsoventral and antero-posterior body axes formation, and regionalization (Figure 2D). Impaired tissue movements during gastrulation, as observed in various LOFs (Figure 2B and Video S1), was prefigured by a strong enrichment for cell migration-associated genes. The genes suppressed or unaffected by the selected signals and maternal TFs were enriched for the ZGA-critical biological processes of mRNA metabolism and translation. For instance, the transient activation of the entire zinc finger cluster (Figure S2A) was not affected by any tested LOF. Because family members are frequently cross-regulated, and the MBT-staged chromatin contains many Krüppel-like zinc finger “footprints” (Gentsch et al., 2018b), it is conceivable that the unaffected, tissue-nonspecific part of ZGA is regulated by maternal zinc finger TFs. This vertebrate gene regulatory branch may be more ancient than that of Pou5F/SoxB1 as zinc finger TFs like Zelda are also key to ZGA of the invertebrate *Drosophila* (Liang et al., 2008).

Video S1. Quadruple LOF of Zygotic T-box TFs, Related to Figure 2 Simultaneous filming of the vegetal (top row) and animal (bottom row) hemisphere of 4x zT LOF (labeled as T-box KD in the video) (left) and control (right) embryos from early gastrula to mid-tail bud stage.

Next, signal-dependent ZGA was resolved in time and space based on (1) the profiling of RNAPII-engaged chromatin from the 32-cell stage to the MBT and (2) known gene expression patterns along the animal-vegetal and dorsoventral axes (Blitz et al., 2017) (Figures 2E and S2B–S2F). In line with the nuclear translocation of their signal mediators (Figure 2A), Wnt, Nodal, and BMP proved to be required for gene activation in different spatiotemporal domains of the early embryo: β-catenin was needed for ∼87% and ∼46% of genes preferentially expressed on the dorsal side and in the VH/MZ, respectively. Some of its target genes like *nodal3.1* and *sia2* were already active by the 32-cell stage (Figures 1C and 2E). On Wnt LOF, the early transcriptional down-regulation was followed by the mis-regulation of opposing cell fate specifiers: the up-regulation of ventral genes (e.g., *id2*, *szl*) and the down-regulation of dorsal genes (e.g., *chrd*, *otx2*). These observations suggest that β-catenin protects dorsal cells from ventralization (Figures 2E and S2C).

Along similar lines, Nodal LOF embryos predominantly displayed a down-regulation of dorsal (∼63%) and VH/MZ-specific (∼73%) genes, although there was no effect of Nodal LOF on the earliest-activated genes at the 32-cell stage or on opposing cell fate regulators (Figures S2A and S2B). Among the first genes to be sensitive to Nodal LOF was the MZ-specific FGF ligand *fgf20*, activated by the 128-cell stage (Figures 1C, S2A, and S2B). By contrast, BMP LOF caused a decrease in ventrally expressed gene expression (∼45%) from the 1,024-cell stage onward (Figures S2A and S2C).

As a comparison, the ubiquitous expression of maternal Pou5f3/Sox3 was required for transcription in all spatiotemporal domains, including, for example, the uniform expression of miR-427 (Figures S2A and S2D). The requirement for Pou5f3/Sox3 was more marked, however, for region-specific genes, in particular those expressed within animal- (∼55%) and ventral-specific (∼67%) domains (Figures S2A and S2D). The maternal TF VegT promoted vegetal identity by activating ∼40% of genes transcriptionally enriched within its own expression domain, the vegetal hemisphere, while suppressing genes that are expressed in the animal hemisphere like *foxi4.2*. The requirement for VegT was similar in ventral- and dorsal-specific genes (∼31% and ∼30%, respectively).

### Wnt/BMP Synergy Enables Uniform ZGA across the Dorsoventral Axis

**Figure 3:**
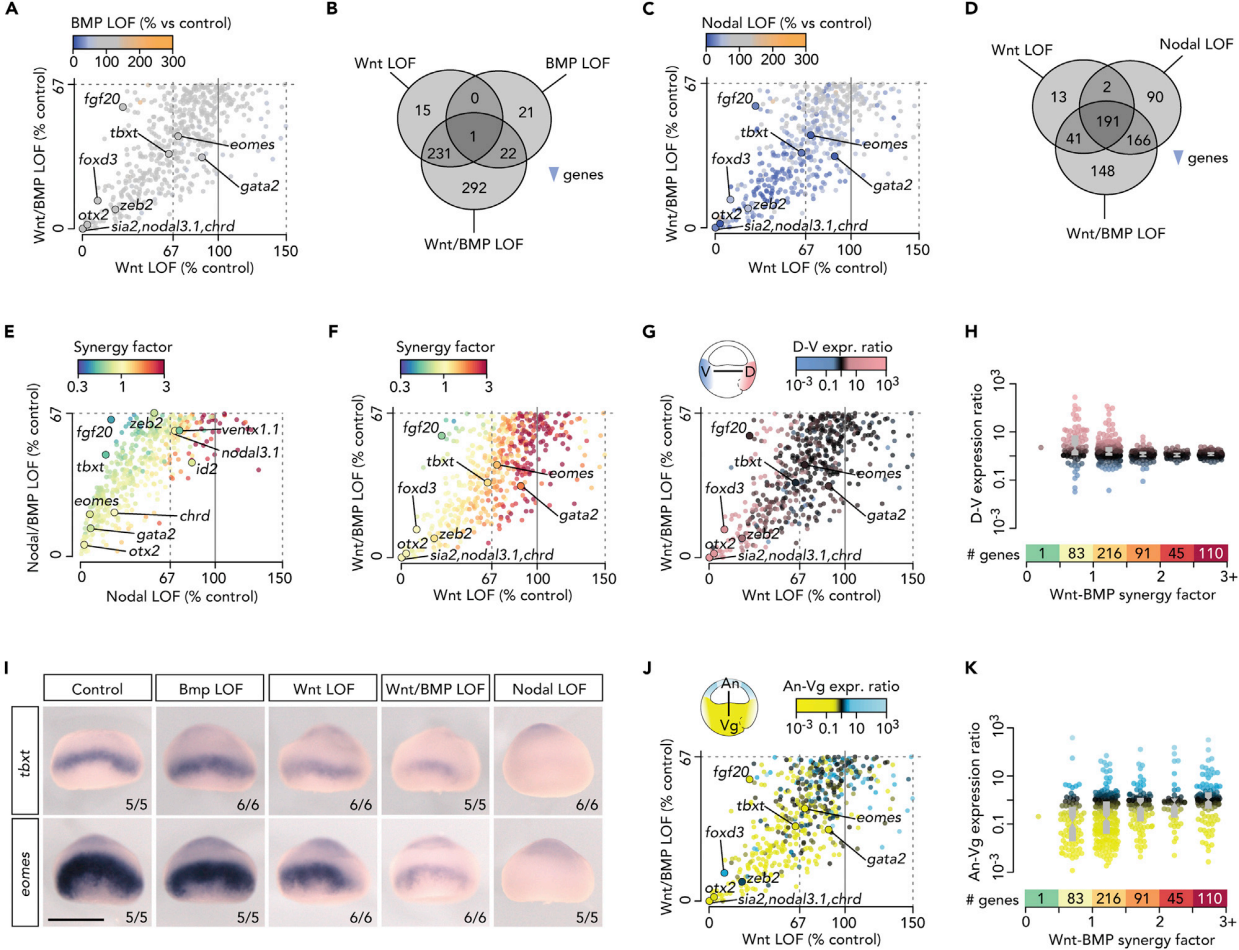
Uniform Gene Expression across Dorsoventral Axis Achieved by Wnt/BMP Synergy (A, C, E, F, G, and J) Scatterplots of relative (% to control) transcript levels between indicated LOFs with each dot (gene) color-coded according to a third attribute: (A and B) relative (% to control) transcript levels, (E and F) synergy factors between single inductive signals, and (G and J) regional expression ratios between opposite ends of the indicated axis. (B and D) Venn diagram of down-regulated genes by indicated LOFs. (H and K) Beeswarm boxplots of regional expression (as measured along the indicated axes) depending on increased Wnt-BMP synergy. (I) Whole-mount in situ hybridization (WMISH) of *tbxt* and *eomes* transcripts under various LOFs. Control and Nodal LOF pictures are from Gentsch et al. (2018b). See also Figure S3 and Table S3.

The relationships between inductive signals with respect to spatial aspects of ZGA were explored by comparing zygotic transcriptomes in single and double LOF experiments. Interestingly, simultaneous loss of both Nodal and BMP function or both Nodal and Wnt caused additive effects on gene expression compared with the single LOFs (Figures 3E and S3A–S3N), whereas simultaneous loss of both BMP and Wnt signaling showed a more synergistic effect (Figures 3A and 3F). These observations are consistent with the morphological consequences of single and double LOFs (Figure 2B). Single LOF experiments revealed very little overlap between Wnt and BMP gene targets (Figure 3B), a result consistent with their domains of activity that are, initially, at opposite ends of the dorsoventral axis. However, dual Wnt/BMP inhibition increased the number of down-regulated genes by 292, a rise of ∼118% and ∼664% with respect to individual Wnt- and BMP-dependent genes, respectively (Figure 3B). Interestingly, this synergy affected 166 Nodal-dependent genes, most of which had uniform expression levels across the dorsoventral axis and differential expression levels across the animal-vegetal axis (Figures 3C, 3D, 3F–3H, 3J, and 3K). Thus, spatially restricted Wnt, BMP, and Nodal signals act together to establish dorsoventral expression uniformity of genes such as *tbxt* and *eomes* (Figure 3I).

**Figure 4:**
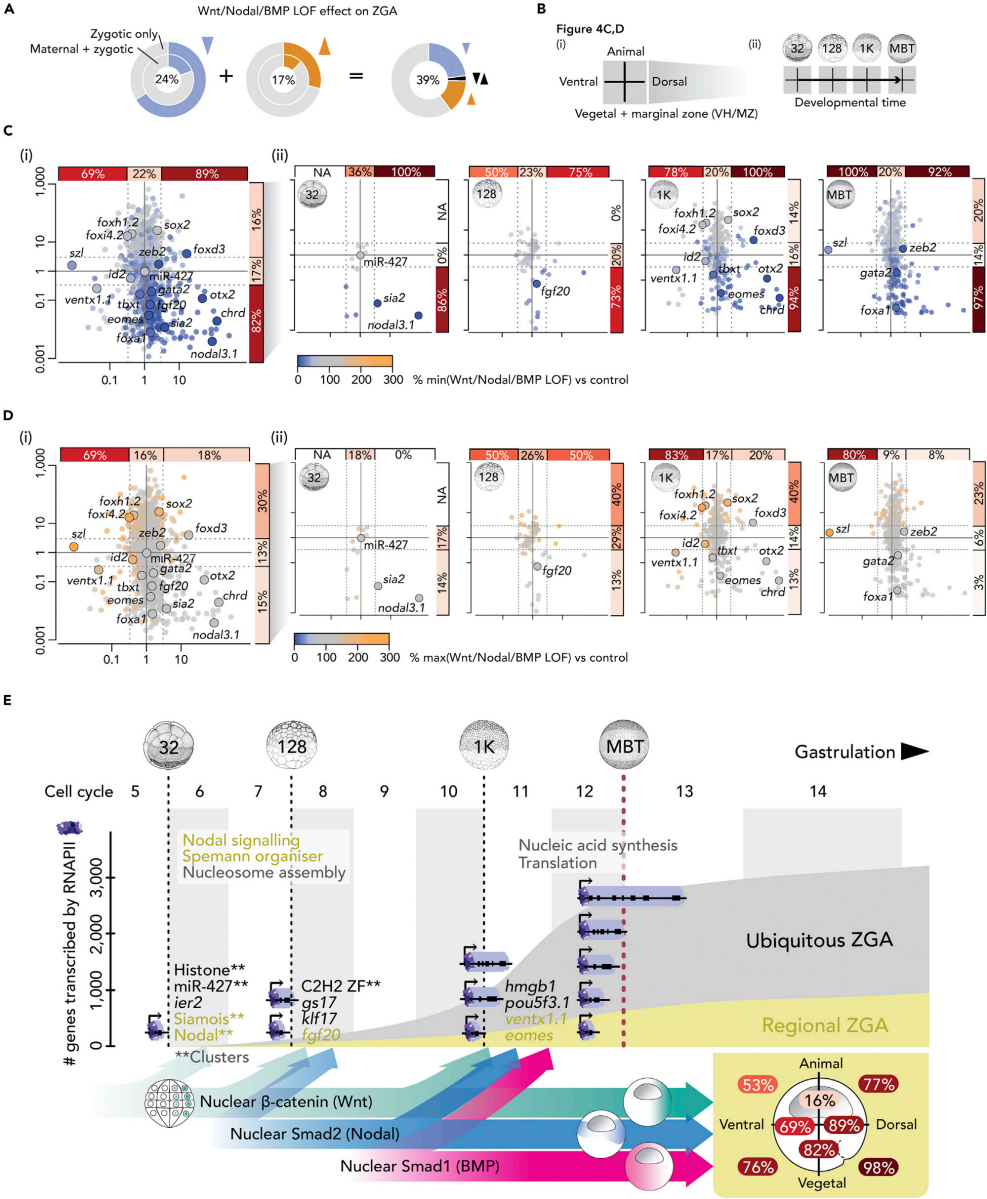
Canonical Wnt, Nodal, and BMP Signals Induce the Majority of Regional ZGA (A) Total percentage of active (zygotic only and maternal-zygotic) genes mis-regulated by Wnt, Nodal, and/or BMP LOF. (B) Graphical explanations of figure panels (C) and (D). (C and D) Summary (i) and temporal resolution (ii) of minimal (C) and maximal (D) transcript levels of active genes (separated by regional expression along the primary body axes) detected among Wnt, Nodal, and/or BMP LOFs. Percentages only refer to the down-regulated (C) or up-regulated (D) genes among all zygotic genes with the same range of expression ratios along the animal-vegetal or dorsoventral axes. (E) Exponential activation of gradually longer genes before the MBT (bulk ZGA) when cell divisions occur at rapid and nearly constant intervals (∼20 min at 28°C). Sequential induction of the canonical Wnt, Nodal, and BMP pathway is critical to high percentages of regional ZGA (as measured along the two primary body axes within the indicated halves and quadrants of an early gastrula embryo): e.g., ∼89% or ∼98% of gene expression enriched in the dorsal half or vegetal-dorsal quadrant, respectively.

Overall, the loss of canonical Wnt, Nodal, and/or BMP signaling caused the mis-regulation of ∼39% (∼22.1% down, ∼2.1% down/up, and ∼14.4% up) of genes activated at ZGA (Figure 4A). These signals were required for most regional ZGA on the dorsal side (∼89%) and in the VH and MZ (∼82%). Notably, their input affected virtually all genes (∼98%, 56/57) with enriched expression in the dorso-vegetal/MZ quadrant (Figures 4B and 4C). Thus, Wnt, Nodal, and BMP substantially contribute to regional ZGA in most anatomical domains of the early gastrula embryo with the exception of animally enriched transcription (∼19%). Animal- and ventral-specific gene expression relies strongly on both activation by ubiquitous maternal TFs (e.g., Pou5f3/Sox3) and on repression by signals (Figures 4B and 4D) and other maternal TFs (e.g., VegT) on the opposite side (Figure S2F).

## Discussion

Our study provides two major insights into the mechanisms by which ZGA is initiated in time and space in *X. tropicalis*. The first concerns the temporal aspects, where we find that RNAPII can be detected across gene bodies well before the MBT, during the period when rapid synchronous cell divisions divide the zygote into 4,096 blastomeres (Figure 4E). The average length of genes covered by RNAPII grows during this time, from ∼1 kb at the 32-cell stage to ∼6 kb at the 1,024-cell stage; these figures compare with an average size of ∼16 kb for maternally expressed genes and for genes expressed after the MBT. Recent long-read sequencing of the zebrafish transcriptome at pre-MBT stages identified transcripts as long as 8 kb spanning multiple pri-miR-430 elements (Nudelman et al., 2018). Furthermore, RNAPII elongation in pre-MBT *Drosophila* embryos occurred at rates of 2.4–3.0 kb/min (Chen et al., 2013, Fukaya et al., 2017).

We do not know why RNAPII, despite its high abundance and its ability to promote rapid elongation, is restricted at early stages from transcribing more genes and longer genes. It may, perhaps, be a consequence of the gradual nature of the chromatin remodeling that occurs during these stages, from the accessibility of *cis*-regulatory elements (Gentsch et al., 2018b, Liu et al., 2018, Lu et al., 2016, Wu et al., 2016) to the spatial organization of an initially unstructured or highly variable chromatin landscape (Du et al., 2017, Flyamer et al., 2017, Hug et al., 2017, Kaaij et al., 2018, Ke et al., 2017). Whatever the reason, the increase of elongated RNAPII engagement between the 128-cell stage and the MBT indicates that a significant component of ZGA disregards the N/C ratio that was originally thought to underlie the onset of transcription at the MBT (Newport and Kirschner, 1982b). Similar conclusions have been drawn from profiling the zygotic transcriptome of haploid *Drosophila* (Lu et al., 2009) and cell-cycle-arrested zebrafish (Chan et al., 2018). Thus, it is becoming clear from work in flies, fish, and frogs that ZGA starts before the MBT and accelerates thereafter (Ali-Murthy et al., 2013, Collart et al., 2014, Mathavan et al., 2005, Owens et al., 2016, Tan et al., 2013), reaching a peak at the MBT (reviewed by Jukam et al., 2017 and Langley et al., 2014).

These observations notwithstanding, it remains possible that cell cycles do contribute to the temporal progression of ZGA and the exponential increase in the number of activated genes before the MBT. In particular, cell cycles may accelerate chromatin remodeling by displacing suppressors in mitotic chromatin and providing unique access to TFs (Halley-Stott et al., 2014) and structural proteins of high-order chromatin (Ke et al., 2017). For example, maternal core histones have been shown to prevent premature ZGA by competing with specific TFs (Joseph et al., 2017).

In addition to the small sizes of the earliest activated genes, we observed that most of these genes, which have no or few introns, code for groups of related factors like histones or zinc finger TFs and that they appear as clusters spanning up to several hundred kilobases. This is in line with previous findings of the earliest active multicopy and intron-poor genes like miR-427 and *nodal5/6* in *Xenopus* embryos (Collart et al., 2014, Lund et al., 2009, Owens et al., 2016, Skirkanich et al., 2011, Takahashi et al., 2006, Yanai et al., 2011, Yang et al., 2002) and miR-430 in zebrafish and Medaka fish (Giraldez et al., 2005, Heyn et al., 2014, Tani et al., 2010). The number and spatial proximity of clustered genes enhances transcriptional output by allowing the sharing of multiple cis-regulatory elements (arranged as super-enhancers) (Whyte et al., 2013) and by fortifying transcriptional condensates of TFs, coactivators, and RNAPII (Boija et al., 2018, Cho et al., 2018, Chong et al., 2018, Sabari et al., 2018, Shrinivas et al., 2018). Overall, based on enriched gene functions, we discovered that ZGA exerts a temporal control of gene expression from nucleosome remodeling before the MBT to protein degradation after the MBT.

Our second insight concerns spatial ZGA and the observation that we can assign a large proportion of spatiotemporal ZGA to key signaling pathways (reviewed by Arnold and Robertson, 2009 and Kimelman, 2006). Canonical Wnt, Nodal, and BMP signaling govern regional ZGA in line with the nuclear translocation of their signal mediators (Faure et al., 2000, Larabell et al., 1997, Schohl and Fagotto, 2002). Thus, Nodal signaling predominantly affects transcription within the vegetal hemisphere and marginal zone, whereas Wnt and BMP initiate transcription in dorsal and ventral regions, respectively. The timing of regional ZGA is defined by the sequential translocation of signal mediators such that nuclear β-catenin directs regional ZGA at the 32-cell stage, followed by nuclear Smad2 at the 128-cell stage and Smad1 at the 1,024-cell stage. Although Smad2-mediated signal transduction depends on the zygotic transcription of its six Nodal ligands (Faure et al., 2000, Gentsch et al., 2018b, Jones et al., 1995, Yang et al., 2002), canonical Wnt and BMP signaling are initiated by the maternally inherited ligands Wnt11 and BMP2/4/7, respectively (Faure et al., 2000, Heasman, 2006, Tao et al., 2005).

We also show that the synergy of opposing signals of the Wnt and BMP pathway affects many Nodal-dependent genes with uniform expression along the dorsoventral axis, such as *eomes* and *Brachyury* (*tbxt*). It is not yet clear whether Wnt/BMP synergy arises from joint chromatin engagement or from mutual or post-translational interactions. For instance, Wnt8a signal can enhance BMP transcriptional readouts by inhibiting the phosphorylation of GSK3, which normally targets Smad1 for degradation (Fuentealba et al., 2007). However, the analysis of *Brachyury* gene regulation in zebrafish suggests that Wnt and BMP can be integrated at a single cis-regulatory DNA element and together with a separate Nodal-responsive DNA element they can establish uniform dorsoventral expression (Harvey et al., 2010). This is further corroborated by our analysis of genome-wide chromatin engagement (Gentsch et al., 2018b): the canonical DNA recognition motif for the Wnt-associated basic helix-span-helix (bHSH) TF AP-2 was more enriched at Smad1 than at Smad2 binding sites (Figure S3O).

We therefore propose that Wnt, BMP, and Nodal signal mediators are critical to regional ZGA and that they balance initially opposing cell fate commitments. However, we have previously shown that signal integration also relies on maternal pioneer TFs like Pou5f3 and Sox3 to make signal-responsive *cis*-regulatory elements accessible for signal mediator binding. For example, Nodal-induced transcription of the *Brachyury* gene depends on the pioneering roles of maternal Pou5f3 and Sox3, and less on their transcriptional activities (Gentsch et al., 2018b).

Overall, we demonstrate that the temporal and spatial dynamics of regional ZGA are regulated by the sequential and spatially restricted translocation of Wnt, Nodal, and BMP signal mediators. These events establish the formation of the primary body axes and germ layers of the embryo. Temporal RNAPII profiling indicates that >1,000 genes of increasing length are activated before MBT and that this substantial portion of ZGA is independent of both the classic N/C ratio and of cell cycle lengthening.

### Limitations of the Study

We detected a dramatic increase in genome-wide recruitment of RNAPII over the cleavage stages during which the genome begins to be transcribed. We used within-sample normalization to scale developmental stage-specific RNAPII profiles. However, because of the large differences in total RNAPII enrichment between samples, chromatin spike-ins are now considered a more accurate method to normalize ChIP-seq profiles across consecutive developmental stages (Chen et al., 2015). We combined separate whole-embryo determinations of RNAPII engagement and transcript levels to reveal the temporal dynamics of ZGA. This approach could be improved by profiling RNAPII-associated RNA to directly couple RNAPII elongation with transcript accumulation (e.g., Churchman and Weissman, 2011). In addition, the spatial resolution of ZGA, which is based on transcriptomics of dissected embryonic parts in our study, could be enhanced by various deep single-cell profiling and super-resolution imaging technologies. We show that most regional ZGA depends on Wnt, Nodal, and BMP signals, but an important question remains: How are these signals integrated at the chromatin level to sustain RNAPII-mediated transcript elongation? In part, this could be investigated by targeted genome editing to increase our understanding of signal-responsive gene regulatory DNA.

## Methods

All methods can be found in the accompanying Transparent Methods supplemental file.

## Supplemental Information

Document S1. Transparent Methods, Figures S1–S3, and Table S1

Table S2. Temporal Progression of ZGA, Related to Figure 1 Activated genes are listed according to the earliest developmental stage of full-length RNAPII occupancy. The lists also include the overall enrichment of RNAPII across the gene body, the maternal contribution (TPM) (Owens et al., 2016), and the enrichment of de novo synthesized transcripts determined by 4sU tagging at the MBT and the early gastrula stage.

Table S3. Differential ZGA Analysis, Related to Figures 2, 3, and 4 Differential expression analysis of genes showing ≥50% (FDR ≤10%) reduced transcript levels in α-amanitin-injected embryos. Normalized transcript levels (inferred from exon or intron counts) are scaled (percentage, %) to the expression level in control embryos. The list also includes FDRs, expression ratios across the animal-vegetal or dorsoventral axis (Blitz et al., 2017), the earliest developmental stage of full-length RNAPII occupancy, and average expression levels between 0 and 1 hpf (maternal) and 5 and 9 hpf (from the MBT to the mid-gastrula stage).
