# Supplementary material for: The Spatiotemporal Control of Zygotic Genome Activation
Source: iScience. 2019 Jun 11;16:485–98. doi: 10.1016/j.isci.2019.06.013 (PMC6593175; doi:10.1016/j.isci.2019.06.013)
Supplement: Document S1. Transparent Methods, Figures S1–S3, and Table S1 [file mmc1.pdf]

ISCI, Volume *16*

Supplemental Information

The Spatiotemporal Control of Zygotic Genome Activation

George E. Gentsch, Nick D.L. Owens, and James C. Smith

SUPPLEMENTAL FIGURES

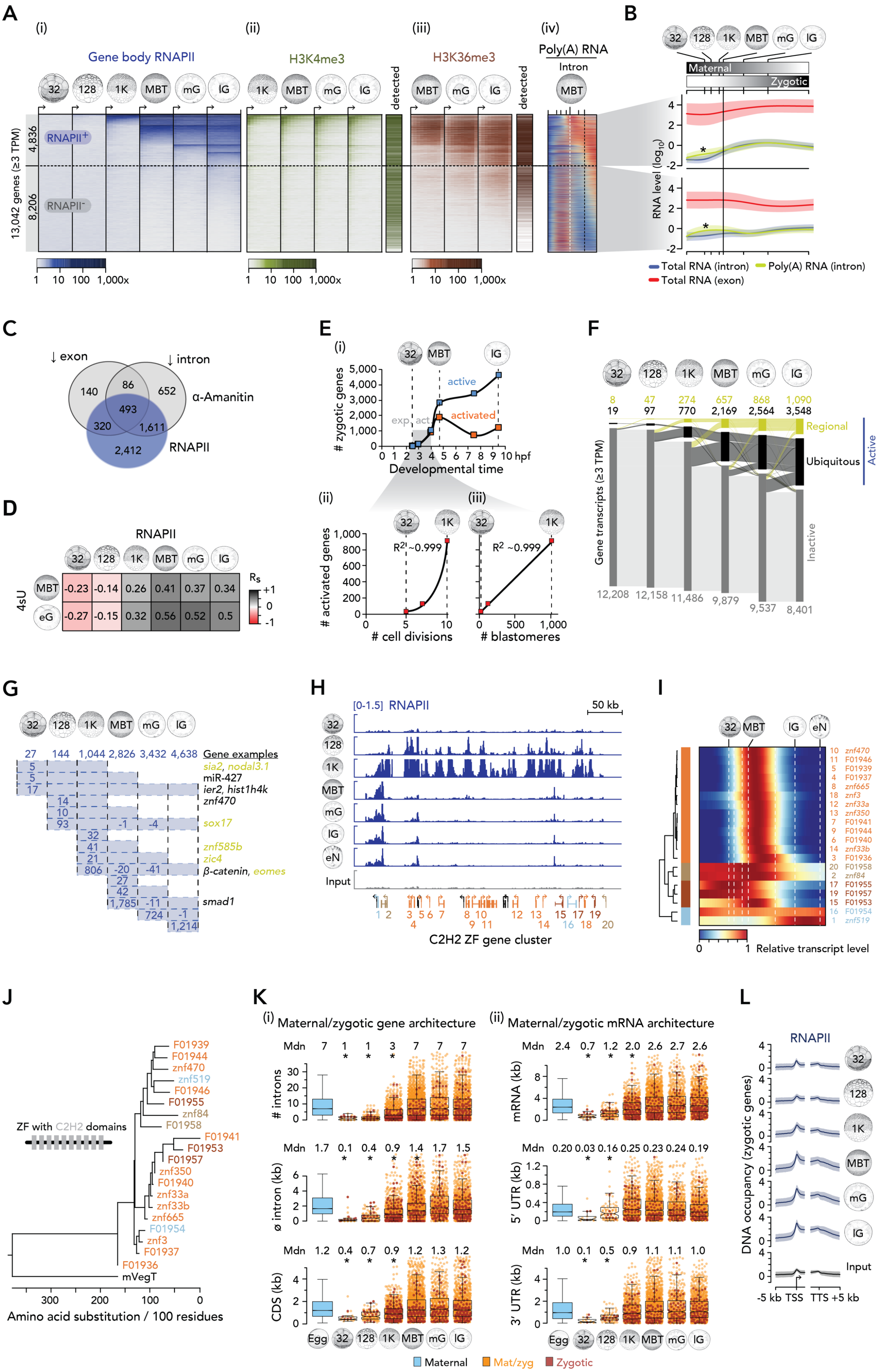

Figure S1. Dynamics and Architecture of ZGA in *X. tropicalis*, Related to Figure 1.

(A) Progression of ZGA from the 32-cell to the late gastrula stage based on (i) whole gene body (full-length) occupancy of RNAPII (i.e., RNAPII was enriched across entire gene bodies; see Transparent Methods). Co-aligned: Active histone marks H3K4me1 (ii) and H3K36me3 (iii) (Hontelez et al., 2015) and intronic signal from the high time-resolution profile of poly(A) RNA (iv) (Owens et al., 2016). The horizontal dotted line separates RNAPII-engaged ($RNAPII^{+}$) from non-engaged ($RNAPII^{-}$) genes as detected until the late gastrula stage. The vertical dotted lines in the poly(A) RNA plot indicate the developmental time points of the MBT (white) and the late gastrula stage (black), respectively.

(B) Transcript feature levels (mean +/- SD) during the maternal-to-zygotic transition. Asterisk, polyadenylation immediately after fertilization (Collart et al., 2014) transiently increased the intronic signal obtained from the poly(A) RNA samples.

(C) Venn diagram of zygotic genes detected by full-length RNAPII occupancy or reduced exonic or intronic transcript counts upon blocking RNAPII-mediated transcription with α-amanitin.

(D) Pairwise Spearman's correlations (R_s) of enrichment values resulting from RNAPII profiling and 4sU tagging to detect zygotic genes at indicated developmental stages.

(E) Plots of the number of active and newly activated genes (i) or newly activated genes versus the developmental time (i), the number of completed cell divisions (ii) or formed blastomeres (iii).

(F) Alluvial diagram of spatio-temporal ZGA including maternally inherited RNA transcripts of genes not activated by the late gastrula stage. Tissue-specificity inferred from regional transcript enrichments along the animal-vegetal or the dorso-ventral or both axes (Blitz et al., 2017).

(G) Numbers of genes with full-length RNAPII occupancy at indicated developmental stages. Examples of ubiquitously (black) and tissue-specifically (orange) expressed genes are listed to the right.

(H) RNAPII dynamics at the Cys2-His2 [C2H2] zinc finger (ZF) cluster from the 32-cell to the early neurula stage.

(I) Expression dynamics of C2H2 ZF genes normalized to maximal transcript levels recorded between fertilization and 23.5 hpf (Owens et al., 2016).

(J) Phylogenetic tree of the C2H2 ZF genes shown in (H,I). Maternal VegT (mVegT), outgroup TF of this phylogenetic tree.

(K) Beeswarm boxplots showing various metrics of the zygotic/maternal genes (i) and mRNA (ii) during ZGA. Asterisks, significant Wilcoxon rank-sum tests against maternal and post-MBT activated genes and corresponding effect sizes (r_{effect}): # introns, $p < 2.1e-13$, r_{effect} 0.07-0.43; Ø intron (kb), $p < 7.9e-7$, r_{effect} 0.07-0.42; CDS (kb), $p < 3e-7$, r_{effect} 0.05-0.27; mRNA (kb), $p < 7e-11$, r_{effect} 0.06-0.32; 5' UTR (kb), $p < 0.015$, r_{effect} 0.03-0.15; and 3' UTR (kb), $p < 1.4e-6$, r_{effect} 0.04-0.21.

(L) Meta-profiles (mean +/- SD) of RNAPII (separated by developmental stage) and input (negative control) densities at zygotic genes.

Abbreviations: 32, 32-cell; 128, 128-cell; 1K, 1,024-cell; MBT, mid-blastula transition; mG, mid-gastrula; lG, late gastrula; eN, early neurula; 4sU, 4-thiouridine; Mdn, median; TPM, transcripts per million.

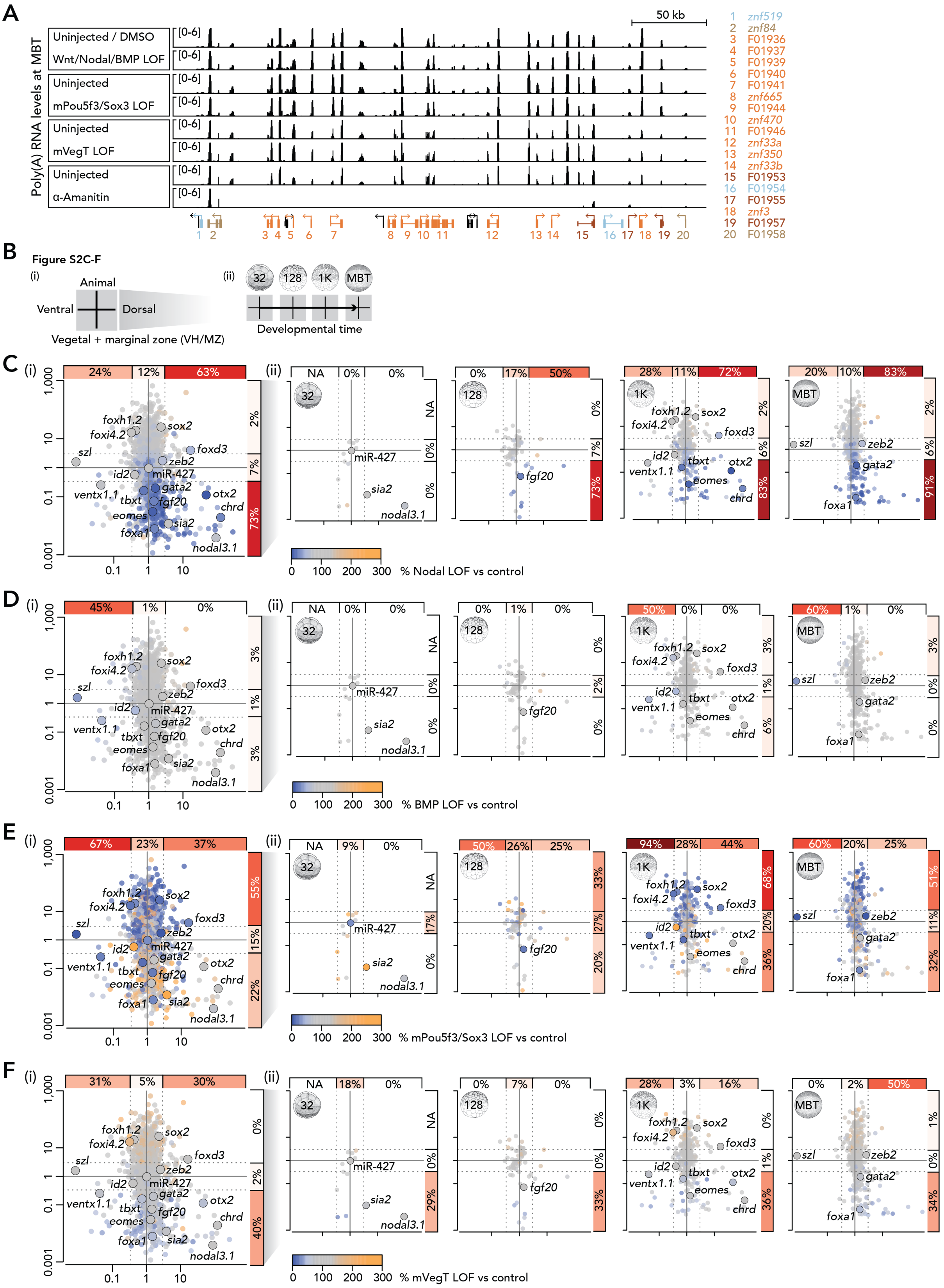

Figure S2. Effect of Canonical Wnt, Nodal and BMP Signals on ZGA, Related to Figure 2.

(A) Poly(A) RNA profiles of the C2H2 ZF cluster (Figure S1H) for indicated control and LOFs at the MBT.

(B) Graphical explanations of figure panels (C-F).

(C-F) Summary (i) and temporal resolution (ii) of gene mis-regulations upon the LOF of Nodal (C) or BMP (D) signaling or maternal Pou5f3/Sox3 (mPou5f3/Sox3) (E) or VegT (mVegT) (F). Percentages only refer to the down-regulated genes (by ≥1/3 compared to control expression level) among all zygotic genes with the same range of expression ratios along the animal-vegetal or the dorso-ventral axes.

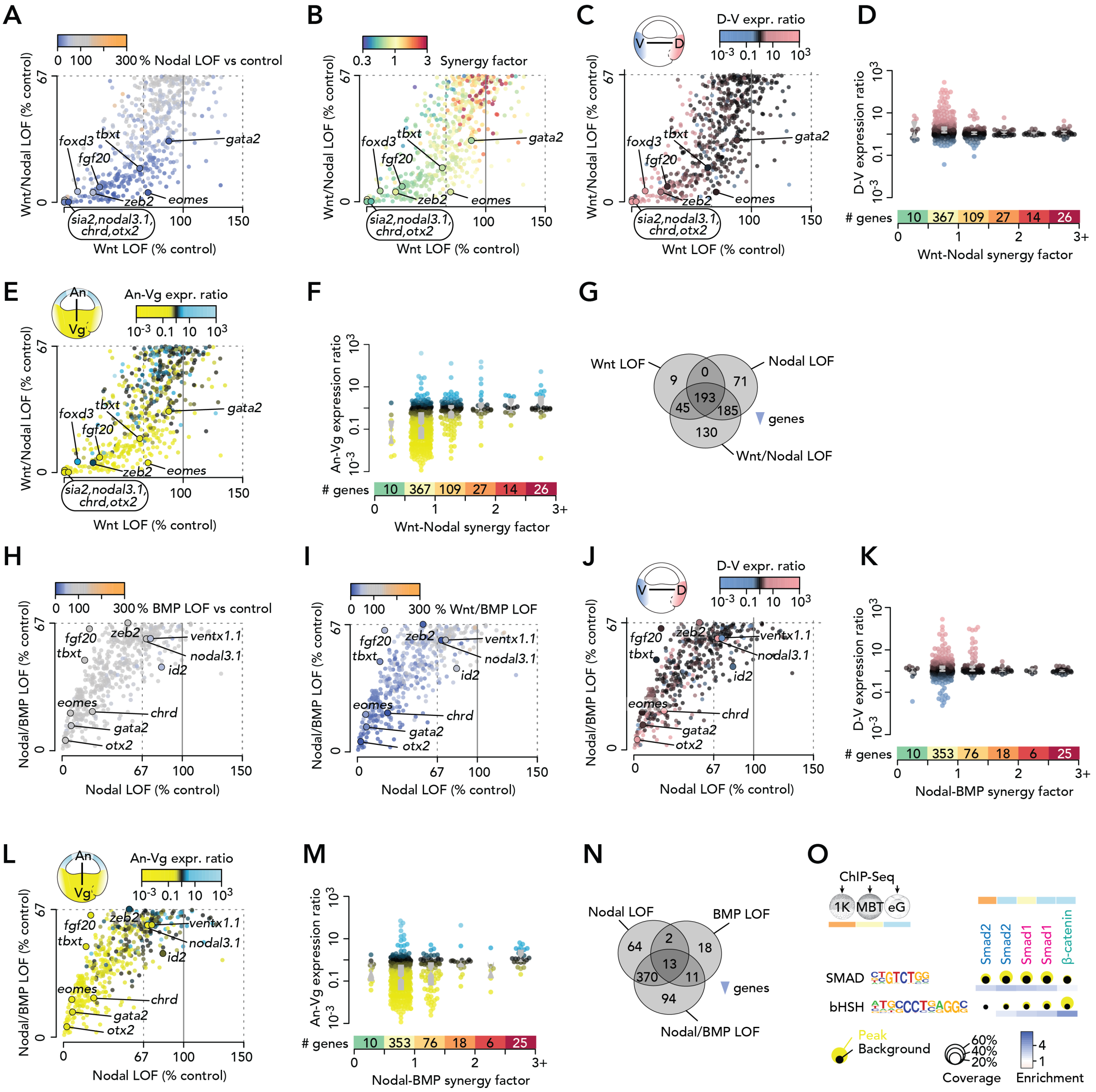

Figure S3. Relationship between Canonical Wnt, Nodal and BMP to Control Regional ZGA, Related to Figure 3.

(A-C,E,H-J,L) Scatter plots of relative (% to control) transcript levels between indicated LOFs with each dot (gene) color-coded according to a third attribute: (A,H,I) relative (% to control) transcript levels, (B) synergy factors between single inductive signals, and (C,E,J,L) regional expression ratios between opposite ends of the indicated axis.

(G,N) Venn diagram of down-regulated genes by indicated LOFs.

(D,F,K,M) Beeswarm boxplots of regional expression (as measured along the indicated axes) depending on increased Wnt-Nodal (D,F) or Nodal-BMP (K,M) synergy.

(O) Coverage and enrichment of Smad and β-catenin-associated DNA motifs (SMAD and bHSH motifs)

at endogenous binding sites of β-catenin, Smad1, Smad2 (Gentsch et al., 2018b) at indicated developmental stages (color-coded).

SUPPLEMENTAL TABLES

Table S1. Summary of Deep sequencing and Read Alignments, Related to Figure 1 and 2.

The meta-data of ChIP-Seq, 4sU-Seq and RNA-Seq runs includes the developmental stage, condition, read type, and total and genome-aligned read numbers. *, all rows represent manually collected biological sample: a, b and c mark biological replicates. †, library was sequenced twice. Reads were pooled from both sequencing runs. ¶, reported numbers are reads that are non-redundantly aligned to the genome assembly 7.1 with a mapping quality (MAPQ) of 10 (DNA) and 255 (RNA). NF, staging according to Nieuwkoop and Faber (1994). PE/SExx, paired-end or single-end sequencing plus read length.

Sample*	Library	Stage	NF	Condition	PE/SExx	Total reads	Aligned reads¶
**1†**	RNAPII ChIP	32-cell	6	None	SE50	45,073,468	25,451,597
**2†**	RNAPII ChIP	128-cell	7	None	SE50/100	65,757,131	41,352,403
**3†**	RNAPII ChIP	1,024-cell	8	None	SE50	60,445,294	37,661,064
**4**	RNAPII ChIP	MBT	8.5	None	SE50	29,196,397	18,402,411
**5**	RNAPII ChIP	mid-gastrula	11	None	SE50	29,268,648	20,085,357
**6†**	RNAPII ChIP	late gastrula	12+	None	SE50	95,959,570	67,591,256
**7**	RNAPII ChIP	early neurula	13+	None	SE50	47,039,656	32,889,013
**8**	4sU-tagged total RNA	Mid-blastula	8+	4sU (4-thiouridine)	PE50	22,202,216	2,738,397
**9**	4sU-tagged total RNA	Early gastrula	10+	4sU (4-thiouridine)	PE50	20,388,030	5,212,782
**10**	Total RNA	Mid-blastula	8+	4sU (4-thiouridine), input	PE50	15,087,016	11,451,545
**11**	Total RNA	Early gastrula	10+	4sU (4-thiouridine), input	PE50	14,902,023	9,771,572
**12a**	Poly(A)+ RNA	Late blastula	9+	Wnt LOF (β-catenin MO)	PE76	39,439,343	35,540,116
**12b**	Poly(A)+ RNA	Late blastula	9+	Wnt LOF (β-catenin MO)	PE76	53,523,555	48,364,696
**12c**	Poly(A)+ RNA	Late blastula	9+	Wnt LOF (β-catenin MO)	PE76	50,568,196	45,534,243
**13a**	Poly(A)+ RNA	Late blastula	9+	Wnt/Nodal LOF (β-catenin MO, SB431542)	PE76	43,980,513	39,732,439
**13b**	Poly(A)+ RNA	Late blastula	9+	Wnt/Nodal LOF (β-catenin MO, SB431542)	PE76	44,582,821	40,373,045
**13c**	Poly(A)+ RNA	Late blastula	9+	Wnt/Nodal LOF (β-catenin MO, SB431542)	PE76	37,243,519	33,648,196
**14a**	Poly(A)+ RNA	Late blastula	9+	Wnt/BMP LOF (β-catenin MO, LDN193189)	PE76	48,440,801	44,080,313
**14b**	Poly(A)+ RNA	Late blastula	9+	Wnt/BMP LOF (β-catenin MO, LDN193189)	PE76	40,989,331	37,188,245
**14c**	Poly(A)+ RNA	Late blastula	9+	Wnt/BMP LOF (β-catenin MO, LDN193189)	PE76	42,494,161	38,527,873
**15a**	Poly(A)+ RNA	Late blastula	9+	Wnt/Nodal/BMP LOF (β-catenin MO, SB431542, LDN193189)	PE76	52,761,134	47,815,313
**15b**	Poly(A)+ RNA	Late blastula	9+	Wnt/Nodal/BMP LOF (β-catenin MO, SB431542, LDN193189)	PE76	55,128,794	50,165,817
**15c**	Poly(A)+ RNA	Late blastula	9+	Wnt/Nodal/BMP LOF (β-catenin MO, SB431542, LDN193189)	PE76	57,414,981	52,101,210
**16a**	Poly(A)+ RNA	Late blastula	9+	Control (uninjected, DMSO)	PE76	42,248,469	37,989,215
**16b**	Poly(A)+ RNA	Late blastula	9+	Control (uninjected, DMSO)	PE76	52,066,825	47,105,847
**16c**	Poly(A)+ RNA	Late blastula	9+	Control (uninjected, DMSO)	PE76	42,875,722	38,893,706
**17a**	Poly(A)+ RNA	Late blastula	9+	Nodal LOF (SB431542)	PE76	47,946,077	43,367,783

**17b**	Poly(A)+ RNA	Late blastula	9+	Nodal LOF (SB431542)	PE76	43,201,587	39,270,622
**17c**	Poly(A)+ RNA	Late blastula	9+	Nodal LOF (SB431542)	PE76	47,497,608	43,105,868
**18a**	Poly(A)+ RNA	Late blastula	9+	Bmp LOF (LDN193189)	PE76	49,171,585	44,430,698
**18b**	Poly(A)+ RNA	Late blastula	9+	Bmp LOF (LDN193189)	PE76	35,247,462	31,937,946
**18c**	Poly(A)+ RNA	Late blastula	9+	Bmp LOF (LDN193189)	PE76	40,645,238	36,899,368
**19a**	Poly(A)+ RNA	Late blastula	9+	Nodal/BMP LOF (SB431542, LDN193189)	PE76	49,865,847	45,127,683
**19b**	Poly(A)+ RNA	Late blastula	9+	Nodal/BMP LOF (SB431542, LDN193189)	PE76	48,875,533	44,167,262
**19c**	Poly(A)+ RNA	Late blastula	9+	Nodal/BMP LOF (SB431542, LDN193189)	PE76	49,397,754	44,710,470
**20a**	Poly(A)+ RNA	Early gastrula	10+	4x zT (4 zygotic T-box TFs) LOF (zVegT, Eomes, Tbxt, Tbxt.2 MO)	PE76	70,597,621	63,789,737
**20b**	Poly(A)+ RNA	Early gastrula	10+	4x zT (4 zygotic T-box TFs) LOF (zVegT, Eomes, Tbxt, Tbxt.2 MO)	PE76	62,453,340	56,799,027
**20c**	Poly(A)+ RNA	Early gastrula	10+	4x zT (4 zygotic T-box TFs) LOF (zVegT, Eomes, Tbxt, Tbxt.2 MO)	PE76	66,378,689	60,011,884
**20d**	Poly(A)+ RNA	Early gastrula	10+	4x zT (4 zygotic T-box TFs) LOF (zVegT, Eomes, Tbxt, Tbxt.2 MO)	PE76	53,693,870	48,621,416
**21a**	Poly(A)+ RNA	Mid-gastrula	11+	4x zT (4 zygotic T-box TFs) LOF (zVegT, Eomes, Tbxt, Tbxt.2 MO)	PE76	60,071,724	53,786,168
**21b**	Poly(A)+ RNA	Mid-gastrula	11+	4x zT (4 zygotic T-box TFs) LOF (zVegT, Eomes, Tbxt, Tbxt.2 MO)	PE76	49,856,218	44,780,174
**21c**	Poly(A)+ RNA	Mid-gastrula	11+	4x zT (4 zygotic T-box TFs) LOF (zVegT, Eomes, Tbxt, Tbxt.2 MO)	PE76	57,420,101	51,441,091
**21d**	Poly(A)+ RNA	Mid-gastrula	11+	4x zT (4 zygotic T-box TFs) LOF (zVegT, Eomes, Tbxt, Tbxt.2 MO)	PE76	57,421,959	51,207,446
**22a**	Poly(A)+ RNA	Late gastrula	12+	4x zT (4 zygotic T-box TFs) LOF (zVegT, Eomes, Tbxt, Tbxt.2 MO)	PE76	59,905,689	53,050,487
**22b**	Poly(A)+ RNA	Late gastrula	12+	4x zT (4 zygotic T-box TFs) LOF (zVegT, Eomes, Tbxt, Tbxt.2 MO)	PE76	76,564,052	67,801,662
**22c**	Poly(A)+ RNA	Late gastrula	12+	4x zT (4 zygotic T-box TFs) LOF (zVegT, Eomes, Tbxt, Tbxt.2 MO)	PE76	54,924,184	48,697,757
**22d**	Poly(A)+ RNA	Late gastrula	12+	4x zT (4 zygotic T-box TFs) LOF (zVegT, Eomes, Tbxt, Tbxt.2 MO)	PE76	56,546,178	49,962,419
**23a**	Poly(A)+ RNA	Early gastrula	10+	Control (standard control MO)	PE76	57,881,388	52,311,548
**23b**	Poly(A)+ RNA	Early gastrula	10+	Control (standard control MO)	PE76	49,193,836	44,649,709
**23c**	Poly(A)+ RNA	Early gastrula	10+	Control (standard control MO)	PE76	78,605,062	70,838,519
**23d**	Poly(A)+ RNA	Early gastrula	10+	Control (standard control MO)	PE76	67,143,066	60,397,172
**24a**	Poly(A)+ RNA	Mid-gastrula	11+	Control (standard control MO)	PE76	50,011,338	44,604,698
**24b**	Poly(A)+ RNA	Mid-gastrula	11+	Control (standard control MO)	PE76	54,847,378	48,981,425
**24c**	Poly(A)+ RNA	Mid-gastrula	11+	Control (standard control MO)	PE76	56,633,663	50,510,544
**24d**	Poly(A)+ RNA	Mid-gastrula	11+	Control (standard control MO)	PE76	69,504,876	61,831,845
**25a**	Poly(A)+ RNA	Late gastrula	12+	Control (standard control MO)	PE76	53,566,350	47,558,871
**25b**	Poly(A)+ RNA	Late gastrula	12+	Control (standard control MO)	PE76	36,043,121	31,918,744
**25c**	Poly(A)+ RNA	Late gastrula	12+	Control (standard control MO)	PE76	48,184,154	42,775,598
**25d**	Poly(A)+ RNA	Late gastrula	12+	Control (standard control MO)	PE76	39,860,708	35,323,477
**Total**						2,948,234,870	2,524,336,789

TRANSPARENT METHODS

CONTACT FOR REAGENTS AND RESOURCE SHARING

Further information and requests for resources and reagents should be directed to and will be fulfilled by the Lead Contact, James C. Smith (jim.smith@crick.ac.uk).

EXPERIMENTAL MODEL AND SUBJECT DETAILS

Xenopus tropicalis Manipulation

Standard procedures were used for ovulation, fertilization, and manipulation and incubation of embryos (Khokha et al., 2002; Sive et al., 2000). Briefly, frogs were obtained from Nasco (Wisconsin, USA). Ovulation was induced by injecting serum gonadotropin (Intervet) and chorionic gonadotropin (Intervet) into the dorsal lymph sac of mature female frogs. Eggs were fertilized *in vitro* with sperm solution consisting of 90% Leibovitz's L-15 medium (Thermo Fisher Scientific, Cat#11415064) and 10% fetal bovine serum (Thermo Fisher Scientific, Cat#10500056). After 10 min, fertilized eggs were de-jellied with 2.2% (w/v) L-cysteine (Merck, Cat#168149) equilibrated to pH 8.0. Embryos were cultured in 5% Marc's Modified Ringer's solution (MMR) (5 mM NaCl, 0.1 mM KCl, 0.1 mM $CaCl_2$, 0.05 mM $MgSO_4$ and 0.25 mM HEPES pH7.5) at 21ºC-28ºC. Embryos were staged according to Nieuwkoop and Faber (1994). All *Xenopus* work fully complied with the UK Animals (Scientific Procedures) Act 1986 as implemented by the Francis Crick Institute.

Chromatin immunoprecipitation (ChIP)

ChIP was carried out as detailed previously (Gentsch and Smith, 2017). Briefly, de-jellied *X. tropicalis* embryos were fixed at room temperature with 1% formaldehyde (Merck, Cat#F8775) in 1% MMR for 25 min. The fixation time was extended to 45 min for pre-gastrula stages. The following number of embryos were used for ChIP-Seq: 1,400 at the 32-cell stage, 1,000 at the 128-cell stage, 700 at the 1,024-cell stage, 450 at the MBT and 350 for the post-MBT stages. Fixation was terminated by rinsing embryos three times with ice-cold 1% MMR. Fixed embryos were homogenized in CEWB1 (150 mM NaCl, 1 mM EDTA, 1% (v/v) Igepal CA-630 [Merck, Cat#I3021], 0.25% (w/v) sodium deoxycholate [Merck, Cat#SRE0046], 0.1% (w/v) sodium dodecyl sulfate [Merck, Cat#71729] and 10 mM Tris-HCl pH 8.0) supplemented with 0.5 mM DL-Dithiothreitol (Fluorochem, Cat#M02712) and protease inhibitors (Roche, Cat#11873580001). The homogenate was left on ice for 5 min and then centrifuged at 1,000 g (4ºC) for 5 min. Homogenization and centrifugation was repeated once before resuspending the pellet in 1-3 ml CEWB1. Chromatin was solubilized and fragmented by microtip-mediated ultra-sonication (Misonix 3000 sonicator with a tapered 1/16-inch microtip). The solution of fragmented chromatin was cleared by centrifuging at 16,000 g (4ºC) for 5 min. About 1% of the cleared chromatin extract was set aside for the input sample (negative control). The remaining chromatin was incubated overnight at 4ºC on a vertical rotor (10 rpm) with 20 µl of the mouse monoclonal anti-RNAPII (8WG16) (Covance, Cat#MMS-126R; RRID: AB_10013665) antibody. After adding 100 µl of washed protein G magnetic beads (Thermo Fisher Scientific, Cat#10003D) the solution was incubated for another 4 h at 4ºC on a vertical rotor (10 rpm). The beads were washed eight times in CEWB1 and once in TEN (10 mM Tris-HCl pH 8.0, 150 mM NaCl and 1 mM EDTA) at 4ºC. ChIP was eluted off the beads twice with 100 µl SDS elution buffer (50 mM Tris-HCl pH 8.0, 1 mM EDTA and 1% (w/v) sodium dodecyl sulfate) at 65ºC. ChIP eluates were pooled before reversing DNA-protein cross-links. Input (filled up to 200 µl with SDS elution buffer) and ChIP samples were supplemented with 10 µl 5 M NaCl and incubated at 65ºC for 6-16 h. Samples were treated with proteinase K (Thermo Fisher Scientific, Cat#AM2548) and RNase A (Thermo Fisher Scientific, Cat#12091021) to remove any proteins and RNA from the co-immunoprecipitated DNA fragments. The DNA was purified with phenol:chloroform:isoamyl alcohol (25:24:1, pH 7.9) (Thermo Fisher Scientific, Cat#AM9730) using 2.0-ml Phase Lock Gel Heavy microcentrifuge tubes (VWR, Cat#733-2478) for phase separation and precipitated with 1/70 volume of 5 M NaCl, 2 volumes of absolute ethanol and 15 µg GlycoBlue (Thermo Fisher Scientific, Cat#AM9516).

After centrifugation, the DNA pellet was air-dried and dissolved in 11 µl elution buffer (10 mM Tris-HCl pH 8.5). The DNA concentration was determined on a Qubit fluorometer using high-sensitivity reagents for detecting double-stranded DNA (10 pg/µl to 100 ng/µl) (Thermo Fisher Scientific, Cat#Q33231).

ChIP-Seq Library Preparation

Using the KAPA Hyper Prep Kit (Roche, Cat#KK8504), 2.5-5 ng ChIP DNA or 5 ng input DNA were converted into indexed paired-end libraries as previously described (Gentsch and Smith, 2017). Briefly, DNA fragments were end-repaired and A-tailed for 30 min at 20ºC followed by 30 min at 65ºC before cooling to 4ºC. 7.5 pmol TruSeq (single index) Y-adapters (IDT) were ligated to the DNA fragment ends for 20 min at 20ºC. The DNA ligation product was extracted with 0.8x SPRI (solid phase reversible immobilisation) beads (Beckman Coulter, Cat#A63882) and amplified in five PCR cycles (15 sec at 98ºC, 30 sec at 60ºC and 30 sec at 72ºC) using the KAPA high-fidelity polymerase master mix (Roche, Cat#KK2602) and 25 pmol Illumina P5 (forward) and P7 (reverse) primers (IDT). After cleaning up the PCR reaction with 1x SPRI beads, the DNA library was size-separated by electrophoresis using E-gel EX agarose gels (Thermo Fisher Scientific, Cat#G401002). A gel slice containing DNA ranging from 250 to 450 bp in size was dissolved shaking in 350 µl QG buffer (Qiagen) using a thermomixer (1,000 rpm) at room temperature. The DNA was purified with MinElute columns (Qiagen, Cat#28604) and eluted off these columns twice using 11 µl elution buffer (10 mM Tris-HCl pH 8.5). The library was re-amplified using another 6-8 PCR cycles yielding 100-200 ng DNA without adapter dimer contamination. The DNA library was cleaned up with 1x SPRI beads.

Illumina Sequencing

All sequencing libraries were quality controlled: The DNA yield and fragment size distribution were determined by fluorometry and chip-based capillary electrophoresis, respectively. ChIP-Seq and RNA-Seq libraries were sequenced on the Illumina HiSeq 2500 and 4000, respectively, by the Advanced Sequencing Facility of the Francis Crick Institute. Sequencing samples and read alignment results are summarized in Table S1.

Post-Sequencing Analysis of ChIP-Seq

Single reads of maximal 50 bases were processed using trim_galore v0.4.2 (Babraham Institute, UK) to trim off low-quality bases (default Phred score of 20, i.e. error probability was 0.01) and adapter contamination from the 3' end. Processed reads were aligned to the *X. tropicalis* genome assembly v7.1 and v9.1 (for Hilbert curves) running Bowtie2 v2.2.9 (Langmead and Salzberg, 2012) with default settings (Table S1). Alignments were converted to the HOMER's tag density format (Heinz et al., 2010) with redundant reads being removed (makeTagDirectory -single -tbp 1 -unique -mapq 10 -fragLength 175 -totalReads all). Only uniquely aligned reads (i.e. MAPQ ≥10) were processed. We pooled all input alignments from various developmental stages (Gentsch et al., 2018b). This created a comprehensive mappability profile that covered ~400 million unique base pair positions. For Hilbert curves, tag densities were generated across the genome v9.1 using sliding (200-bp increments) 400-bp window. Background signals (<0.3 reads per 1 million mapped reads) were removed. Blacklisted (Gentsch et al., 2018b) regions

(except for MIR-427) were excluded using intersectBed (-v -f 0.5) from BEDtools v2.25.0 (Quinlan and Hall, 2010).

Detecting Zygotic and Maternal Genes Using RNAPII Profiling and High Time-Resolution Transcriptomics

Normalized RNAPII and input tag densities were calculated across the gene body in 10 bins of equal size. Gene annotations v7.1 were altered based on a few known zygotic isoforms and some corrections obtained from assembling total and poly(A) RNA (Owens et al., 2016) from stage 6 to stage 12.5 *de novo* (Pertea et al., 2016). A few genes had previously been annotated as gene clusters due to assembly uncertainties. We reduced the annotation of polycistronic MIR-427 to the minus arm (scaffold_3b:3516900-3523400) and only monitored *nodal3.5* and *nodal5.3* within their respective gene clusters. Gene bodies with <40% mappability were removed. Here, the threshold of mappability per bin was set at 10% of the input read density averaged across all gene bodies in use. Subsequently, enrichment values were only obtained for all mappable bins by dividing read densities of RNAPII and input. Further, we restricted the analysis to genes for which ≥3 transcripts per million (TPM) could be detected on average over three consecutive time points (i.e. over the developmental time of 1 h) of a high-resolution profile of total RNA (Owens et al., 2016) from fertilization to after gastrulation (stage 13). Genes were considered active when RNAPII enrichments along their full length (see thresholds below) and corresponding transcripts (≥0.1 TPM) were simultaneously detected. Transcript levels were calculated over three consecutive time points +/- 1 h from the developmental stage of RNAPII profiling. RNAPII enrichment covered ≥80% of the mappable gene body and reached at least one of the following thresholds: (1) 2.6-fold, (2) 1.8-fold and 1.4-fold at the next or previous stage, (3) 1.4-fold and 1.8-fold at the next or previous stage, or (4) 1.4-fold over three consecutive stages. The heatmap (Figure 1B and S1A) was sorted by the developmental stage (1st) and the overall fold (2nd) of RNAPII enrichment. Zygotic and maternal contributions to transcriptome (Figure 1G) were based on RNAPII enrichment (see above) and mean transcript levels (≥0.1 TPM) detected between 0 and 1 hpf, respectively.

Peak Calling and Motif Enrichment Analysis

Peak calling and motif enrichment analysis were carried out as previously reported (Gentsch et al., 2018b). Briefly, HOMER v4.8.3 (Heinz et al., 2010) was used to identify the binding sites of Smad1 (Gentsch et al., 2018b), Smad2 (Chiu et al., 2014; Gentsch et al., 2018b; Yoon et al., 2011) and β-catenin (Gentsch et al., 2018b; Nakamura et al., 2016) by virtue of ChIP-enriched read alignments (hereafter called peaks): findpeaks -style factor -minDist 175 -fragLength 175 -inputFragLength 175 -fdr 0.001 -gsize 1.435e9 -F 3 -L 1 -C 0.97. This means that both ChIP and input alignments were extended 3’ to 175 bp for the detection of significant (FDR ≤0.1%) peaks being separated by ≥175 bp. The effective size of the *X. tropicalis* genome assembly v7.1 was set to 1.435 billion bp, an estimate obtained from the mappability profile (Gentsch et al., 2018b). These peaks showed equal or higher tag density than the surrounding 10 kb, ≥3-fold more tags than the input and ≥0.97 unique tag positions relative to the expected number of tags. To further eliminate any false positive peaks, we removed any peaks with <0.5 CPM and those falling into blacklisted regions showing equivocal mappability due to genome assembly errors, gaps or simple/tandem repeats. Regions of equivocal mappability were identified by a two-fold lower (poor) or three-fold higher

(excessive) read coverage than the average detected in 400-bp windows sliding at 200-bp intervals through normalized ChIP input and DNase-digested naked genomic DNA (Gentsch et al., 2018b). All identified regions ≤800 bp apart were subsequently merged. Gap coordinates were obtained from the Francis Crick mirror site of the UCSC genome browser (http://genomes.crick.ac.uk). Simple repeats were masked with RepeatMasker v4.0.6 (Smit et al.) using the crossmatch search engine v1.090518 (Phil Green) and the following settings: RepeatMasker -species "xenopus silurana tropicalis" -s -xsmall. Tandem repeats were masked with Jim Kent's trfBig wrapper script of the Tandem Repeat Finder v4.09 (Benson, 1999) using the following settings: weight for match, 2; weight for mismatch, 7; delta, 7; matching probability, 80; indel probability, 10; minimal alignment score, 50; maximum period size, 2,000; and longest tandem repeat array (-l), 2 [million bp]. The enrichment and occurrence of predetermined DNA binding motifs was calculated using 100 bp centred across the top 2,000 peaks per chromatin feature and developmental stage: findMotifsGenome.pl -size 100 -mknown -nomotif.

Injections and Treatments of Embryos

Microinjections were performed using calibrated needles and embryos equilibrated in 4% (w/v) Ficoll PM-400 (Merck, Cat#F4375) in 5% MMR. Microinjection needles were generated from borosilicate glass capillaries (Harvard Apparatus, GC120-15) using the micropipette puller Sutter p97. Maximally three nanolitres were injected into the animal hemisphere of de-jellied zygotes using the microinjector Narishige IM-300. Embryos were transferred to fresh 5% MMR (without Ficoll PM-400) once they reached about the mid-blastula stage.

For profiling the nascent transcriptome, embryos were injected with 75 ng 4-thiouridine-5'-triphosphate (4sU) (TriLink BioTechnologies, Cat#N-1025), which is incorporated into newly synthesized transcripts.

Loss-of-functions (LOFs) were generated by treating embryos with small molecule inhibitors and/or injecting them with morpholinos (MOs) or α-amanitin. MOs were designed and produced by Gene Tools (Oregon, USA) to block splicing (MO_{splice}) or translation (MO_{transl}): maternal Pou5f3/Sox3 (mPou5f3/Sox3) LOF, 5 ng *Pou5f3.2* MO_{transl} (Chiu et al., 2014; Gentsch et al., 2018b; GCTGTTGGCTGTACATAGTGTC), 5 ng *Pou5f3.3* MO_{transl} (Chiu et al., 2014; Gentsch et al., 2018b; TACATTGGGTGCAGGGACCCTCTCA) and 5 ng *Sox3* MO_{transl} (Gentsch et al., 2018b; GTCTGTGTCCAACATGCTATACATC); maternal VegT (mVegT) LOF, 10 ng m*VegT* MO_{transl} (Gentsch et al., 2018b; Rana et al., 2006; TGTGTTCCTGACAGCAGTTTCTCAT); canonical Wnt LOF, 5 ng *β-catenin* MO_{transl} (Heasman et al., 2000; TTTCAACAGTTTCCAAAGAACCAGG); LOF of four zygotic T-box TFs (4x zT LOF), 2.5 ng *tbxt (Xbra, t)* MO_{splice} (Gentsch et al., 2013; TGGAGAGACCCTGATCTTACCTTCC), 2.5 ng *tbxt (Xbra, t)* MO_{transl} (Gentsch et al., 2013; GGCTTCCAAGCGCACACACTGGG), 2.5 ng *tbxt.2 (Xbra3, t2)* MO_{splice} (Gentsch et al., 2013; GAAAGGTCCATATTCTCTTACCTTC), 2.5 ng *tbxt.2* (*Xbra3*, *t2*) MO_{transl} (Gentsch et al., 2013; AGCTGTGCCTGTGCTCATTGTATTG), 5 ng z*VegT* MO_{transl} (Fukuda et al., 2010; Gentsch et al., 2013; CATCCGGCAGAGAGTGCATGTTCCT) and 5 ng *eomes* MO_{splice} (Fukuda et al., 2010; Gentsch et al., 2013; GAACATCCTCCTGCAAAGCAAAGAC); control MO, 5-20 ng standard control MO (CCTCTTACCTCAGTTACAATTTATA) according to the dose used for the β-catenin, mVegT and 4x zT LOF experiment; and 30 pg α-amanitin (BioChemica, Cat#A14850001). To block Nodal (Nodal LOF)

and BMP (BMP LOF) signaling, embryos were treated with 100 µM SB431542 (Tocris, Cat#1614) and/or 10 µM LDN193189 (Selleckchem, Cat#S2618) from the 8-cell stage onwards. Control embryos were treated accordingly with DMSO, in which these antagonists were dissolved. Transcriptional effects of combinatorial signal LOF were determined at late blastula stage (stage 9^+), while those of all other maternal LOFs were determined over three consecutive time points: the MBT (stage 8+), the late blastula (stage 9^+) and the early gastrula (stage 10^+) stage. The 4x zT LOF was transcriptionally profiled at early, mid and late gastrula stage (stage 10^+, 11^+ and 12^+). The 4x zT LOF comparison has four biological replicates (n=4). All other comparisons entail three biological replicates (n=3).

Extraction of Total RNA

Embryos were homogenized in 800 µl TRIzol reagent (Thermo Fisher Scientific, Cat#15596018) by vortexing. The homogenate was either snap-frozen in liquid nitrogen and stored at -80ºC or processed immediately. For phase separation, the homogenate together with 0.2x volume of chloroform was transferred to pre-spun 2.0-ml Phase Lock Gel Heavy microcentrifuge tubes (VWR), shaken vigorously for 15 sec, left on the bench for 2 min and spun at ~16,000 g (4ºC) for 5 min. The upper phase was mixed well with one volume of 95-100% ethanol and spun through the columns of the RNA Clean & Concentrator 25 Kit (Zymo Research, Cat#R1017) at ~12,000 g for 30 sec. Next, the manufacturer's instructions were followed for the recovery of total RNA (>17 nt) with minor modifications. First, the flow-through of the first spin was re-applied to the column. Second, the RNA was treated in-column with 3 U Turbo DNase (Thermo Fisher Scientific, Cat#AM2238). Third, the RNA was eluted twice with 25 µl molecular-grade water. The concentration was determined on the NanoDrop 1000 spectrophotometer or by fluorometry before depleting ribosomal RNA from total RNA (Profiling the Nascent Transcriptome).

Tagging the Nascent Transcriptome

Thirty 4sU-injected embryos were collected at the MBT and the early-to-mid gastrula stage. Total RNA was extracted as outlined above. The 4sU-tagging was performed according to Gay et al. (2014) with few minor modifications. The RNA Clean & Concentrator 5 Kit (Zymo Research, Cat#R1013) was used to purify RNA. Briefly, the Ribo-Zero Gold rRNA Removal Kit (Illumina, Cat#MRZG126) was used according to the manufacturer's instructions to deplete ribosomal RNA from ~10 µg total RNA. The RNA was purified and fragmented for 4 min at 95ºC using the NEBNext Magnesium RNA Fragmentation Module (NEB, Cat#E6150). The RNA was purified again before conjugating HDPD-Biotin (Thermo Fisher Scientific, Cat#21341) to 4sU via disulfide bonds for 3 h in the dark. Purified RNA was mixed with Streptavidin beads (Thermo Fisher Scientific, Cat#65305) to pull down biotin-tagged RNA. The RNA was eluted off the beads by treating them twice with 100 µl pre-heated (80ºC) 100 mM β-mercaptoethanol (Merck, Cat#M6250), which breaks the disulfide bond between Biotin and 4sU. Subsequently, the RNA was converted into a deep sequencing library by following the manual instructions (Rev. C, 8/2014) of the ScriptSeq v2 RNA-Seq Library Preparation Kit (Illumina, Cat#SSV21106) starting with 4.1.A. (Anneal the cDNA Synthesis Primer) and 4.1.B. (Synthesize cDNA), RNA and ending with part 3.C (Synthesize 3'-Tagged DNA) to 3.G. (Assess Library Quantity and Quality). cDNA was purified using 1.8x SPRI beads. Input and 4sU-enriched cDNA were PCR-amplified with 11 and 15 cycles, respectively. The 4sU RNA-Seq library was purified with 1x SPRI beads.

Post-Sequencing Analysis of 4sU Tagging

Paired-end reads were aligned to the *X. tropicalis* transcriptome assembly v7.1 running Bowtie2 (Langmead and Salzberg, 2012) with the following constraints: -k 200 (maximal allowed number of alignments per fragment) -X 800 (maximum fragment length in bp) --rdg 6,5 (penalty for read gaps of length N, 6+N*5) --rfg 6,5 (penalty for reference gaps of length N, 6+N*5) --score-main L,-.6,-.4 (minimal alignment score as a linear function of the read length x, f(x) = -0.6 - 0.4*x) --no-discordant (no paired-end read alignments breaching maximum fragment length X) --no-mixed (only concordant alignment of paired-end reads). Only read pairs that uniquely align to one gene were counted. Raw read counts were normalized with DESeq2 v1.22.1 (Love et al., 2014) and then scaled to the input.

Poly(A) RNA-Seq Profiling

10-15 embryos were collected per stage and condition. Total RNA was extracted as outlined above. Libraries were made from ~1 µg total RNA by following the low-sample protocol of the TruSeq RNA Library Prep Kit v2 (Illumina, Cat#RS-122-2001) with a few modifications. First, 1 µl cDNA purified after second strand synthesis was quantified on a Qubit fluorometer using high-sensitivity reagents for detecting double-stranded DNA (10 pg/µl to 100 ng/µl). By this stage, the yield was ~10 ng. Second, the number of PCR cycles was reduced to eight to avoid products of over-amplification such as chimera fragments.

Poly(A) RNA-Seq Read Alignment

Paired-end reads were aligned to the *X. tropicalis* genome assembly v7.1 using STAR v2.5.3a (Dobin et al., 2013) with default settings. The alignment was guided by a revised version of the gene models v7.2 (Collart et al., 2014) to improve mapping accuracy across splice junctions. The alignments were sorted by read name using the sort function of Samtools v1.3.1 (Li et al., 2009). Exon and intron counts (-t 'exon;intron') were extracted from unstranded (-s 0) alignment files using VERSE v0.1.5 (Zhu et al., 2016) in featureCounts (default) mode (-z 0). Intron coordinates were adjusted to exclude any overlap with exon annotation. For visualization, genomic BAM files of biological replicates were merged using Samtools and converted to the bigWig format. These genome tracks were normalized to the wigsum of 1 billion excluding any reads with mapping quality <10 using the python script bam2wig.py from RSeQC v2.6.4 (Wang et al., 2012).

Differential Gene Expression Analysis

Differential expression analysis was performed with both raw exon and intron counts excluding those belonging to ribosomal and mitochondrial RNA using the Bioconductor/R package DESeq2 v1.22.1 (Love et al., 2014). In an effort to find genes with consistent fold changes over time, p-values were generated according to a likelihood ratio test reflecting the probability of rejecting the reduced (~ developmental stage) over the full (~ developmental stage + condition) model. Resulting p-values were adjusted to obtain false discovery rates (FDR) according to the Benjamini-Hochburg procedure with thresholds on Cook's distances and independent filtering being switched off. Equally, combinatorial LOF profiling and regional expression datasets (Blitz et al., 2017) without time series were subjected to likelihood ratio tests with reduced (~ 1) and full (~ condition) models for statistical analysis. Fold changes of intronic and exonic transcript levels were calculated for each developmental stage and condition using the mean of DESeq2-normalized read counts from biological replicates. Both intronic and exonic

datasets were filtered for ≥10 DESeq2-normalized read counts that were detected at least at one developmental stage in all uninjected or DMSO-treated samples. Gene-specific fold changes were removed at developmental stages that yielded <10 normalized read counts in corresponding control samples. Next, the means of intronic and exonic fold changes were calculated across developmental stages. The whole dataset was confined to 3,318 genes for which at least 50% reductions (FDR ≤10%) in exonic (default) or intronic counts could be detected in α-amanitin-injected embryos. Regional expression was based on exonic read counts by default unless the intronic fold changes were significantly (FDR ≤10%) larger than the exonic fold changes (Table S3). For the hierarchical clustering of relative gene expression (Figure 2C), increased transcript levels were masked and only data points from signal LOFs, mPou5f3/Sox3 LOF and 4x zT LOF embryos were used. Euclidean distance-derived clusters were linked according to Ward's criterion and sorted using the optimal leaf ordering (OLO) algorithm. The synergy factor (SF) between signals x and y (Figures 3 and S3) were calculated as follows: $SF_{xy} = \Delta_{xy} / (\Delta_x + \Delta_y)$. Δ is the relative loss of gene expression caused by signal depletion. For these calculations, any gene upregulations were neutralised (i.e. set to 1).

Analysis of Enriched Gene Ontology (GO) Terms

Over-represented GO terms were found by applying hypergeometric tests of the Bioconductor/R package GOstats v2.42.0 (Falcon and Gentleman, 2007) on gene lists. The process was also supported by the Bioconductor/R packages GSEABase v1.44.0 (Morgan et al., 2017) and GO.db v3.4.1 (Carlson et al., 2007). The gene universe was associated with GO terms by means of BLAST2GO (Conesa et al., 2005) as previously outlined (Collart et al., 2014; Gentsch et al., 2015).

Generation of Hybridization Probes

Plasmids *X. laevis eomes* pCRII-TOPO (Gentsch et al., 2013) and *X. laevis tbxt (Xbra, t)* pSP73 (Smith et al., 1991) were linearized by restriction digestion (*Bam*HI and *Bgl*II, respectively) and purified using the QIAquick PCR Purification Kit (Qiagen, Cat#28104). The hybridization probes were transcribed from ~1 µg linearized plasmid using 1x digoxigenin-11-UTP (Roche, Cat#11277065910), 40 U RiboLock RNase inhibitor (Thermo Fisher Scientific, Cat#EO0381), 1x transcription buffer (Roche) and T7 RNA polymerase (Roche, Cat#10881767001) at 37ºC for 2 h. The probe was treated with 2 U Turbo DNase (Thermo Fisher Scientific) to remove the DNA template and purified by LiCl precipitation. RNA was diluted to 10 ng/µl (10x stock) with hybridization buffer. The hybridization buffer (stored at -20ºC) consists of 50% formamide (Fisher Scientific, Cat#10052370), 5x saline sodium citrate (SSC), 1x Denhardt's solution (Thermo Fisher Scientific, Cat#750018), 10 mM EDTA, 1 mg/ml torula RNA (Merck, Cat#R6625), 100 µg/ml heparin (Merck, Cat#H4784), 0.1% (v/v) Tween-20 (Merck, Cat#P9416) and 0.1% (w/v) CHAPS (Merck, Cat#C3023).

Whole-Mount In Situ Hybridization (WMISH)

WMISH was conducted using digoxigenin-labeled RNA probes (Monsoro-Burq, 2007; Sive et al., 2000). Briefly, *X. tropicalis* embryos were fixed in MEMFA (100 mM MOPS pH 7.4, 2 mM EDTA, 1 mM $MgSO_4$ and 3.7% formaldehyde) at room temperature for 1 h. The embryos were then washed once in 1x PBS and two to three times in ethanol. Fixed and dehydrated embryos were kept at -20ºC for ≥24 h to ensure proper dehydration before starting hybridization. Dehydrated embryos were washed once more in

ethanol before rehydrating them in two steps to PBT (1x PBS and 0.1% (v/v) Tween-20). Embryos were treated with 5 µg/ml proteinase K (Thermo Fisher Scientific) in PBT for 6-8 min, washed briefly in PBT, fixed again in MEMFA for 20 min and washed three times in PBT. Embryos were transferred into baskets, which were kept in an 8x8 microcentrifuge tube holder sitting inside a 10x10 slot plastic box filled with PBT. Baskets were built by replacing the round bottom of 2-ml microcentrifuge tubes with a Sefar Nitex mesh. This container system was used to readily process several batches of embryos at once. These baskets were maximally loaded with 40 to 50 *X. tropicalis* embryos. The microcentrifuge tube holder was used to transfer all baskets at once and to submerge embryos into subsequent buffers of the WMISH protocol. Next, the embryos were incubated in 500 µl hybridization buffer (see recipe above) for 2 h in a hybridization oven set to 60ºC. After this pre-hybridization step, the embryos were transferred into 500 µl digoxigenin-labeled probe (1 ng/µl) preheated to 60ºC and further incubated overnight at 60ºC. The pre-hybridization buffer was kept at 60ºC. The next day embryos were transferred back into the pre-hybridization buffer and incubated at 60ºC for 10 min. Subsequently, they were washed three times in 2x SSC/0.1% Tween-20 at 60ºC for 10 min, twice in 0.2x SSC/0.1% Tween-20 at 60ºC for 20 min and twice in 1x maleic acid buffer (MAB) at room temperature for 5 min. Next, the embryos were treated with blocking solution (2% Blocking Reagent [Merck, Cat#11096176001] in 1x MAB) at room temperature for 30 min, and incubated in antibody solution (10% lamb serum [Thermo Fisher Scientific, Cat#16070096], 2% Blocking Reagent [Merck], 1x MAB and 1:2,000 Fab fragments from polyclonal anti-digoxigenin antibodies conjugated to alkaline phosphatase [Roche, Cat#11093274910; RRID:AB_514497]) at room temperature for 4 h. The embryos were then washed four times in 1x MAB for 10 min before leaving them in 1x MAB overnight at 4ºC.

On the final day of the WMISH protocol, the embryos were washed another three times in 1x MAB for 20 min and equilibrated to working conditions of alkaline phosphatase (AP) for a total of 10 min by submerging embryos twice into AP buffer (50 mM $MgCl_2$, 100 mM NaCl, 100 mM Tris-HCl pH 9.5 and 1% (v/v) Tween-20). At this stage, the embryos were transferred to 5-ml glass vials for monitoring the progression of the AP-catalyzed colorimetric reaction. Any residual AP buffer was discarded before adding 700 µl staining solution (AP buffer, 340 µg/ml nitro-blue tetrazolium chloride [Roche, Cat#11383213001] and 175 µg/ml 5-bromo-4-chloro-3'-indolyphosphate [Roche, Cat#11383221001]). The colorimetric reaction was developed at room temperature in the dark. Once the staining was clear and intense enough, the color reaction was stopped by two washes in 1x MAB. To stabilize and preserve morphological features, the embryos were fixed with Bouin's fixative without picric acid (9% formaldehyde and 5% glacial acetic acid [Fisher Scientific, Cat#10171460]) at room temperature for 30 min. Next, the embryos were washed twice in 70% ethanol/PBT to remove the fixative and residual chromogens. After rehydration to PBT in two steps, the embryos were treated with weak Curis solution (1% (v/v) hydrogen peroxide [Merck, Cat#1072090500], 0.5x SSC and 5% formamide) at 4ºC in the dark overnight. Finally, the embryos were washed twice in PBS before imaging them in PBS on a thick agarose dish by light microscopy.

Processing of External Datasets

High-time (30-min intervals) resolution of total and poly(A) RNA-Seq (GEO: GSE65785) was processed as reported in the original publication (Owens et al., 2016). In addition, intron read counts were corrected by spike RNA-derived normalization factors. For visualization normalized exon and intron counts were scaled to the maximal count detected across the time course and fitted using cubic smoothing splines from 0 to 23.5 hpf: smooth.spline(1:48, x, spar=0.6). Other RNA-Seq (GEO: GSE81458) and ChIP-Seq (GEO: GSE67974, GEO: GSE30146, GEO: GSE53654 and GEO: GSE72657) were processed as described in detail above except for H3K4me3 and H3K36me3 whose enriched regions were detected as follows: findPeaks -style histone -fragLength 175 -inputFragLength 175 -fdr 0.001 -gsize 1.435e9 -F 2 -C 1 -region -size 350 -minDist 500. Thus, we detected significant regions of histone modifications (-style histone) of at least the lengths of two DNA fragments (-size 350) and being separated by at least 500 bp from each other.

Generation of Plots and Heatmaps

Genomic snapshots were generated with the IGV genome browser v2.4-rc6 (Robinson et al., 2011). All plots and heatmaps were generated using R v3.5.1 (http://cran.r-project.org/). The following add-on R and Bioconductor packages were used for sorting and graphical visualization of data: alluvial v0.1-2 (Michał Bojanowsk), beeswarm v0.2.3 (Aron Eklund), circlize v0.4.5 (Gu et al., 2014), complexHeatmap v1.20.0 (Gu et al., 2016a), dplyr v0.7.8, ggplot2 v3.1.0 (Wickham, 2016), gplots v3.0.1 (Gregory Warnes and colleagues), GenomicFeatures v1.34.1 (Lawrence et al., 2013), GenomicRanges v1.38.0 (Lawrence et al., 2013), HilbertCurve v1.12.0 (Gu et al., 2016b), limma v3.38.2 (Ritchie et al., 2015), rtracklayer 1.42.1 (Lawrence et al., 2009) and seriation v1.2-3 (Hahsler et al., 2008).

QUANTIFICATION AND STATISTICAL ANALYSIS

No statistical method was used for determining sample size; rather, we followed the literature to select the appropriate sample size. The experiments were not randomized. Due to the nature of experiments, the authors were not blinded to group allocation during data collection and analysis. Only viable embryos were included in the analysis. Frequencies of shown morphological phenotypes and WMISH patterns are included in every image. The significance of over-represented GO terms was based on hypergeometric tests. Significances of non-normally distributed data points (gene features) across ZGA were calculated using paired Wilcoxon rank-sum tests (alternative hypothesis 'less'). The effect size (r_{effect}) was estimated from the standard normal deviate of the Wilcoxon p-value (p) as previously described (Rosenthal, 1991), r_{effect}=Z/sqrt(N), where Z=qnorm(1-p/2) is the standardized Z-score and N is the number of observations.

For RNA-Seq, biological triplicates were used to account for transcriptional variability between clutches. Each LOF experiment has its own control embryos collected in parallel from the same mothers: exp. #1 (α-amanitin), uninjected embryos; exp. #2 (BMP or Nodal LOF), DMSO-treated embryos; exp. #3 (Wnt LOF), uninjected embryos; exp. #4 (mPou5f3/Sox3 LOF), uninjected embryos; exp. #5 (mVegT LOF), uninjected embryos; exp. #6 (single and combinatorial LOFs of Wnt, Nodal and BMP), DMSO-treated embryos; exp. #7 (combinatorial LOF of 4 zygotic T-box TFs), control MO-injected embryos. The gene expressions of control MO-injected embryos of exp. #2 and #5 were normalized to their corresponding

uninjected embryos. The mean of these normalizations and conservative FDR estimations (i.e., higher FDR of the two likelihood ratio tests) were used for the comparison with LOF conditions. RNA-Seq libraries from each experiment were generated simultaneously to mitigate any batch effects. The FDR was controlled for multiple comparisons according to the Benjamini-Hochberg procedure. The exact computational implementation of differential expression analysis is outlined on GitHub (see below).

DATA AND CODE AVAILABILITY

The accession numbers for the sequencing reads (FASTQ files) and raw RNA-Seq read counts reported in this paper are GEO: GSE113186 and GEO: GSE122551. All analyses were performed in R v3.5.1 (Bioconductor v3.8), Perl v5.18.2 (https://www.perl.org) and Python v2.7.12 (http://www.python.org) as detailed above. The R code, genome annotation, intermediate datasets and graphs are available on GitHub at https://github.com/gegentsch/SpatioTemporalControlZGA. Original datasets are also available on Mendeley Data at http://dx.doi.org/10.17632/jn466b4n8v.1.

SUPPLEMENTAL REFERENCES

Benson, G. (1999). Tandem repeats finder: a program to analyze DNA sequences. Nucleic Acids Res. *27*, 573–580.

Blitz, I.L., Paraiso, K.D., Patrushev, I., Chiu, W.T.Y., Cho, K.W.Y., and Gilchrist, M.J. (2017). A catalog of Xenopus tropicalis transcription factors and their regional expression in the early gastrula stage embryo. Dev. Biol. *426*, 409–417.

Carlson, M., Falcon, S., Pages, H., and Li, N. (2007). Bioconductor - GO.db.

Chiu, W.T., Le, R.C., Blitz, I.L., Fish, M.B., Li, Y., Biesinger, J., Xie, X., and Cho, K.W.Y. (2014). Genome-wide view of TGFβ/Foxh1 regulation of the early mesendoderm program. Development *141*, 1–114.

Collart, C., Owens, N.D.L., Bhaw-Rosun, L., Cooper, B., De Domenico, E., Patrushev, I., Sesay, A.K., Smith, J.N., Smith, J.C., and Gilchrist, M.J. (2014). High-resolution analysis of gene activity during the Xenopus mid-blastula transition. Development *141*, 1927–1939.

Conesa, A., Götz, S., García-Gómez, J.M., Terol, J., Talón, M., and Robles, M. (2005). Blast2GO: a universal tool for annotation, visualization and analysis in functional genomics research. Bioinformatics *21*, 3674–3676.

Dobin, A., Davis, C.A., Schlesinger, F., Drenkow, J., Zaleski, C., Jha, S., Batut, P., Chaisson, M., and Gingeras, T.R. (2013). STAR: ultrafast universal RNA-seq aligner. Bioinformatics *29*, 15–21.

Falcon, S., and Gentleman, R. (2007). Using GOstats to test gene lists for GO term association. Bioinformatics *23*, 257–258.

Fukuda, M., Takahashi, S., Haramoto, Y., Onuma, Y., Kim, Y.-J., Yeo, C.-Y., Ishiura, S., and Asashima, M. (2010). Zygotic VegT is required for Xenopus paraxial mesoderm formation and is regulated by Nodal signaling and Eomesodermin. Int. J. Dev. Biol. *54*, 81–92.

Gay, L., Karfilis, K.V., Miller, M.R., Doe, C.Q., and Stankunas, K. (2014). Applying thiouracil tagging to mouse transcriptome analysis. Nat. Protoc. *9*, 410–420.

Gentsch, G.E., and Smith, J.C. (2017). Efficient Preparation of High-Complexity ChIP-Seq Profiles from Early Xenopus Embryos. Methods Mol. Biol. *1507*, 23–42.

Gentsch, G.E., Owens, N.D.L., Martin, S.R., Piccinelli, P., Faial, T., Trotter, M.W.B., Gilchrist, M.J., and Smith, J.C. (2013). In vivo T-box transcription factor profiling reveals joint regulation of embryonic

neuromesodermal bipotency. Cell Rep. *4*, 1185–1196.

Gentsch, G.E., Patrushev, I., and Smith, J.C. (2015). Genome-wide snapshot of chromatin regulators and states in Xenopus embryos by ChIP-Seq. J. Vis. Exp. *96*, e52535.

Gentsch, G.E., Spruce, T., Monteiro, R.S., Owens, N.D.L., Martin, S.R., and Smith, J.C. (2018a). Innate Immune Response and Off-Target Mis-splicing Are Common Morpholino-Induced Side Effects in Xenopus. Dev. Cell *44*, 597–610.e10.

Gentsch, G.E., Spruce, T., Owens, N.D.L., and Smith, J.C. (2018b). The role of maternal pioneer factors in predefining first zygotic responses to inductive signals. bioRxiv 306803. DOI: https://doi.org/10.1101/306803

Gu, Z. Eils, R., and Schlesner, M. (2016a). Complex heatmaps reveal patterns and correlations in multidimensional genomic data. Bioinformatics *32*, 2847-2849.

Gu, Z., Eils, R., and Schlesner, M. (2016b). HilbertCurve: an R/Bioconductor package for high-resolution visualization of genomic data. Bioinformatics *32*, 2372–2374.

Gu, Z., Gu, L., Eils, R., Schlesner, M., and Brors, B. (2014). circlize Implements and enhances circular visualization in R. Bioinformatics *30*, 2811–2812.

Hahsler, M., Hornik, K., and Buchta, C. (2008). Getting Things in Order: An Introduction to the R Package seriation. J. Stat. Softw. *25*, 1–34.

Heasman, J., Kofron, M., and Wylie, C. (2000). βCatenin Signaling Activity Dissected in the Early Xenopus Embryo: A Novel Antisense Approach. Dev. Biol. *222*, 124–134.

Heinz, S., Benner, C., Spann, N., Bertolino, E., Lin, Y.C., Laslo, P., Cheng, J.X., Murre, C., Singh, H., and Glass, C.K. (2010). Simple combinations of lineage-determining transcription factors prime cis-regulatory elements required for macrophage and B cell identities. Mol. Cell *38*, 576–589.

Hontelez, S., van Kruijsbergen, I., Georgiou, G., van Heeringen, S.J., Bogdanović, O., Lister, R., and Veenstra, G.J.C. (2015). Embryonic transcription is controlled by maternally defined chromatin state. Nat. Commun. *6*, 10148.

Khokha, M., Chung, C., Bustamante, E., Gaw, L., Trott, K., Yeh, J., Lim, N., Lin, J., Taverner, N., Amaya, E., et al. (2002). Techniques and probes for the study of Xenopus tropicalis development. Dev. Dyn. *225*, 499–510.

Langmead, B., and Salzberg, S.L. (2012). Fast gapped-read alignment with Bowtie 2. Nat. Methods *9*, 357–359.

Lawrence, M., Gentleman, R., and Carey, V. (2009). rtracklayer: an R package for interfacing with genome browsers. Bioinformatics *25*, 1841-1842.

Lawrence, M., Huber, W., Pagès, H., Aboyoun, P., Carlson, M., Gentleman, R., Morgan, M., and Carey, V. (2013). Software for Computing and Annotating Genomic Ranges. PLoS Comput. Biol. *9*, e1003118.

Li, H., Handsaker, B., Wysoker, A., Fennell, T., Ruan, J., Homer, N., Marth, G., Abecasis, G., Durbin, R., 1000 Genome Project Data Processing Subgroup (2009). The Sequence Alignment/Map format and SAMtools. Bioinformatics *25*, 2078–2079.

Love, M.I., Huber, W., and Anders, S. (2014). Moderated estimation of fold change and dispersion for RNA-seq data with DESeq2. Genome Biol. *15*, 550.

Monsoro-Burq, A.H. (2007). A Rapid Protocol for Whole-Mount In Situ Hybridization on Xenopus Embryos. Cold Spring Harb. Protoc. *2007*, pdb.prot4809.

Morgan, M., Falcon, S., and Gentleman, R. (2017). *GSEABase: Gene set enrichment data structures*

and methods.

Nakamura, Y., de Paiva Alves, E., Veenstra, G.J.C., and Hoppler, S. (2016). Tissue- and stage-specific Wnt target gene expression is controlled subsequent to β-catenin recruitment to cis-regulatory modules. Development *143*, 1914–1925.

Nieuwkoop, P., and Faber, J. (1994). Normal table of Xenopus laevis (Daudin): a systematical and chronological survey of the development from the fertilized egg till the end of metamorphosis. Garland.

Owens, N.D.L., Blitz, I.L., Lane, M.A., Patrushev, I., Overton, J.D., Gilchrist, M.J., Cho, K.W.Y., and Khokha, M.K. (2016). Measuring Absolute RNA Copy Numbers at High Temporal Resolution Reveals Transcriptome Kinetics in Development. Cell Rep. *14*, 632–647.

Pertea, M., Kim, D., Pertea, G.M., Leek, J.T., and Salzberg, S.L. (2016). Transcript-level expression analysis of RNA-seq experiments with HISAT, StringTie and Ballgown. Nat. Protoc. *11*, 1650–1667.

Quinlan, A.R., and Hall, I.M. (2010). BEDTools: a flexible suite of utilities for comparing genomic features. Bioinformatics *26*, 841–842.

Rana, A., Collart, C., Gilchrist, M., and Smith, J. (2006). Defining synphenotype groups in Xenopus tropicalis by use of antisense morpholino oligonucleotides. PLoS Genet. *2*, e193.

Ritchie, M.E., Phipson, B., Wu, D., Hu, Y., Law, C.W., Shi, W., and Smyth, G.K. (2015). limma powers differential expression analyses for RNA-sequencing and microarray studies. Nucleic Acids Res. *43*, e47.

Robinson, J.T., Thorvaldsdóttir, H., Winckler, W., Guttman, M., Lander, E.S., Getz, G., and Mesirov, J.P. (2011). Integrative genomics viewer. Nat. Biotechnol. *29*, 24–26.

Rosenthal, R. (1991). Meta-Analytic Procedures for Social Research. SAGE Publishing.

Sive, H., Grainger, R., and Harland, R. (2000). Early development of Xenopus laevis: A laboratory manual. Cold Spring Harbor Laboratory Press.

Smit, A., Hubley, R., and Green, P. RepeatMasker Open-4.0. 2013-2015.

Smith, J., Price, B., Green, J., Weigel, D., and Herrmann, B. (1991). Expression of a Xenopus homolog of Brachyury (T) is an immediate-early response to mesoderm induction. Cell *67*, 79–87.

Wang, L., Wang, S., and Li, W. (2012). RSeQC: quality control of RNA-seq experiments. Bioinformatics *28*, 2184–2185.

Wickham, H. (2016). ggplot2: Elegant Graphics for Data Analysis. Springer.

Yoon, S.-J., Wills, A.E., Chuong, E., Gupta, R., and Baker, J.C. (2011). HEB and E2A function as SMAD/FOXH1 cofactors. Genes Dev. *25*, 1654–1661.

Zhu, Q., Fisher, S.A., Shallcross, J., and Kim, J. (2016). VERSE: a versatile and efficient RNA-Seq read counting tool. bioRxiv 053306. DOI: https://doi.org/10.1101/053306
